# Screening to identify people with type 2 diabetes at risk of liver cancer in primary care: a randomised controlled trial protocol

**DOI:** 10.1136/bmjopen-2024-088043

**Published:** 2025-03-06

**Authors:** Ryan M Buchanan, Tina Reinson, Josh Bilson, Hazel Woodland, Chinonso Nwoguh, Keith Cooper, Scott Harris, Karen Malone, Christopher D Byrne

**Affiliations:** 1 University of Southampton Faculty of Medicine, Southampton, UK; 2 University Hospital Southampton NHS Foundation Trust, Southampton, UK; 3 Clinical and Experimental Sciences Division, University of Southampton Faculty of Medicine, Southampton, UK; 4 Salisbury District Hospital NHS Foundation Trust, Salisbury, UK; 5 Southampton Health Technology Assessment Centre, University of Southampton, Southampton, UK; 6 The Old Fire Station Surgery, Woolston, UK; 7 NIHR Southampton Biomedical Research Centre, Southampton, UK

**Keywords:** Randomised Controlled Trial, Hepatobiliary tumours, Health Care Costs, Diabetes & endocrinology, Hepatology

## Abstract

**Introduction:**

Hepatocellular carcinoma (HCC) is expected to become the third most common cause of cancer death worldwide by 2030. The increase in HCC is in large part due to the rising prevalence of risk factors such as type 2 diabetes mellitus (T2DM). Up to 1 in 20 people living with T2DM have liver cirrhosis, and they have a 1% to 2% incidence of HCC per year. Patients with cirrhosis enter surveillance for HCC to identify early-stage, curable tumours. A diagnosis of T2DM does not mandate testing to identify patients with cirrhosis, with testing restricted to those with additional risks. There has never been a trial and nested cost-effectiveness evaluation comparing screening all patients with T2DM for cirrhosis against usual care.

**Methods and analysis:**

The study will use a multi-centre, unblinded individual randomised controlled trial design. The aim will be to determine the effectiveness and cost-effectiveness of screening all adults with T2DM to identify those at high risk of HCC. The recruitment strategy has been supported by patient and public involvement (PPI). Participants will be identified via an automated search of primary care records and invited to participate via text. 320 participants will be randomised for screening. The screening will include measurement of bio-markers for liver fibrosis (ELF and Fib-4) and vibration-controlled transient elastography. Another 320 participants will be randomised to standard care. Demographic and medical history data will be collected at baseline from all participants. Outcome data will be collected remotely from healthcare records. The primary outcome is the proportion of participants in each arm who are referred to HCC surveillance following testing for liver disease within 12 months of randomisation. The results will be used to calculate the incremental cost-effectiveness ratio of screening via a Markov model.

**Ethics and dissemination:**

The results of this study will be presented directly to National Health Service England. Additional dissemination via conference proceedings and publication will be supported by our PPI team. Ethical approval was granted by the West of Scotland Research Ethics Service on 2 August 2023, REC reference 23/WS/0102.

**Trial registration number:**

ISRCTN17017677.

**STRENGTHS AND LIMITATIONS OF THIS STUDY**

- First comparison via a randomised controlled trial between risk factor-based testing for liver disease in people with type 2 diabetes mellitus (T2DM) (usual care in the United Kingdom) and screening offered to all adults with T2DM.
- Provides definitive cost-effectiveness of both approaches and impact on liver cancer diagnosis and survival in a real-world setting.
- Will delineate the relative cost-effectiveness of different non-invasive tests to identify significant liver disease in people with T2DM.
- Trial is limited to the United Kingdom so usual care may not be internationally representative.
- Short study time horizon; therefore, observation of clinical outcomes is subject to modelling rather than real-world observation.

## Introduction

 Cancer is the leading cause of mortality in patients with type 2 diabetes mellitus (T2DM)^1^ and is strongly associated with site-specific cancers including hepatocellular carcinoma (HCC).^2^ 830 200 people died from HCC in 2020, and the incidence of HCC is expected to increase by 55% in the next 20 years.^3^ HCC is now the fastest growing indication for liver transplantation,^4^ and it is expected to become the third most common cause of cancer death worldwide by 2030.^5^ HCC has a very poor prognosis with a 5-year survival of ~20%.^6^ However, if cases are identified at an early stage, curative treatments are available which include surgical resection, liver transplant or tumour ablation.^6^

A major driver for the increasing number of deaths from HCC is the increasing global prevalence of T2DM.^3 5 7^ T2DM causes liver steatosis, inflammation, fibrosis and liver cirrhosis and patients with significant liver fibrosis or cirrhosis are at risk of HCC.^8 9^ There is a high prevalence of all stages of liver disease in people living with T2DM.^10^,^14^

International guidance recommends biannual surveillance for HCC in patients with liver cirrhosis via ultrasound imaging; however, less than one-third of incident cases of HCC in patients with T2DM are identified via surveillance.^15^ Identification of HCC via surveillance is important as cancers that are identified in patients who are undergoing regular surveillance have better outcomes.^16^ To engage patients with T2DM with HCC surveillance, it is necessary to first identify patients with cirrhosis. In the past, liver disease was hard to identify because it progresses without signs or symptoms. However, several approaches have now been validated in patients with T2DM to identify asymptomatic disease. These include the utilisation of blood tests such as the Fibrosis-4 test (FIB-4)^17^ and the Enhanced Liver Fibrosis (ELF) test,^18^ as well as a simple scan of the liver which uses vibration-controlled transient elastography (VCTE) to assess the liver stiffness^1719^,^21^ as a validated marker of fibrosis.

In addition to HCC surveillance, early diagnosis of liver disease can facilitate positive interventions aimed at improving patient outcomes. These include optimisation of blood glucose control in people with T2DM, dietary modification and treatments to facilitate weight loss, moderation or complete abstinence from alcohol (a co-factor in liver disease progression for these patients^22^) and potential pharmacotherapy that reduces fibrogenesis. With respect to the latter, on 14 March 2024, Resmetirom^23^ was given conditional approval by the US Food and Drug Administration for the treatment of adults with non-cirrhotic non-alcoholic steatohepatitis with moderate to advanced liver scarring (fibrosis) alongside diet and exercise. Furthermore, selected patients could be prescribed beta-blocker therapy to reduce mortality from bleeding oesophageal varices and to reduce the risk of liver decompensation.^24^

In addition to being recommended in the USA,^25 26^ screening for liver disease in patients with T2DM and obesity has recently been adopted as a national pilot in England that has been funded by the National Health Service England (NHSE) cancer service.^27^ The national pilot uses a primary care-based search algorithm for T2DM as well as other risk factors for liver disease (such as hazardous alcohol consumption) and then invites patients into a cascade of non-invasive tests for fibrosis.

While patients with T2DM are known to have an increased risk of fibrosis and cirrhosis,^28^ there is a lack of empirical evidence supporting the implementation of this NHSE programme. Just three studies have tested a diagnostic pathway for liver disease against a contemporaneous control,^29^,^31^ and just one specifically focused on liver disease in patients with T2DM.^30^

The NHSE pilot is different from the current national (NICE) guidelines in the UK which recommend testing for liver disease is restricted to patients with risk factors for liver cirrhosis including a fatty liver on ultrasound imaging, abnormal liver enzyme levels and potentially harmful levels of alcohol consumption.^32^ T2DM alone is not a risk factor that currently mandates assessment. The reason for these narrow criteria is a lack of cost-effectiveness data supporting wider eligibility for testing.^33^

The NICE NAFLD guideline (ng49) was published in 2016,^32^ and since its publication, researchers have modelled the cost-effectiveness of testing for liver disease in patients with T2DM.^34^,^36^ Published models have compared testing strategies that include novel biomarkers and VCTE against standard care where standard care includes history, physical examination, liver ‘function’ tests (LFTs) and an ultrasound scan. The sensitivity and specificity of each approach are pre-defined and parameterise models that calculate the health gain for patients correctly categorised with liver disease and offset this against the cost of the different testing approaches by calculating an incremental cost-effectiveness ratio (ICER).^34 36^ Most recently, Forlano *et al* modelled the ICER for screening in patients with T2DM. The model was parameterised using cross-sectional data from a cohort of patients with T2DM living in London (UK) who were all tested for liver cirrhosis using FIB-4, ELF, VCTE and in 19/249 cases, liver biopsy. The costs and outcomes associated with testing this cohort were compared with usual care (primary care diagnosis) which was less accurate. In the base case analysis, the ICER was well below NICE cost-effectiveness thresholds, with the additional costs of testing being offset by the gain from an accurate early diagnosis.

However, there are challenges with extrapolating prior models to a real-world intervention such as the NHSE pilot that aims to test a broader range of adults with T2DM for liver disease as part of routine care. First, we do not know the characteristics of patients who will respond to an invitation from primary care for liver assessment. These characteristics are important—it is likely that patient age and comorbidities will influence their probability of having liver disease and their personal gain from an early diagnosis. Second, we do not know what proportion of this cohort meets clinical criteria for interventions that convey the advantage of early diagnosis, for example, what proportion enter an HCC surveillance pathway and what proportion have clinically significant portal hypertension and are started on beta blockers. Third, we do not know the real-world performance of standard care in the UK. Most patients with T2DM do not get tested for liver disease, despite their heightened risk because they are not assessed for the additional risk factors that are needed to qualify for testing. For example, LFTs are not part of an annual diabetes check-up in the UK and may or may not be measured when patients are considered for statin treatment; liver ultrasound is not a routine test, and alcohol consumption is not accurately or consistently assessed in primary care. Finally, previous economic evaluations are outdated as they use primary-care-based assessments (eg history and examination) that do not incorporate tests for fibrosis (eg Fib-4 and VCTE). Since the NICE NAFLD Guideline in 2016, tests for liver fibrosis have widely integrated into community diagnostic pathways for liver disease and therefore in future studies, models of ‘usual care’ need to reflect this.

This study protocol describes a randomised controlled trial with a nested cost-effectiveness evaluation. The study aims to compare the number of participants referred for HCC surveillance between an intervention where patients with T2DM are universally offered screening for liver disease against usual care.

## Method and analysis

The trial is described in accordance with the SPIRIT checklist.^37^ The design will be an unblinded randomised controlled trial with a nested cost-effectiveness evaluation comparing the offer of screening to *all* patients with T2DM for liver disease against standard care. We will proceed straight to an effectiveness evaluation rather than conducting a formal feasibility/pilot study. We justify this approach because the components of the intervention (used in testing for liver disease in patients with standard risk factors (eg abnormal blood results or harmful alcohol consumption)) are widely implemented. Additionally, data such as the attrition rate from the conventional diagnostic pathway is already known (see sample size section).^38^ Undertaking a randomised controlled trial in this setting is very important as this provides a contemporaneous standard care arm as a counterfactual.

### Primary outcome

The number of participants referred to secondary care with the suspected liver disease within 12 months of randomisation who are subsequently referred for HCC surveillance.

As an unblinded trial, it is important our primary outcome is as objective as possible and independent of the research team. In both study arms, patients with high liver stiffness measurements will be referred to nearby hepatology services (with thresholds defined by local practice). Via usual care, an independent local clinician will then assess the severity of liver disease. In real-world practice, this may include history, examination, no further tests or repeat VCTE, additional tests for fibrosis and in some cases liver biopsy. Regardless of the clinical approach taken, the primary outcome will be whether the clinician felt the disease was severe enough to warrant referral for HCC surveillance. Since the trial sites cover a variety of different regions across the south of England, this pragmatic approach is likely to closely reflect current UK practice.

### Secondary outcomes

1. The test or combination of tests for liver cirrhosis with the lowest cost per case diagnosed.*
2. The sub-group with the lowest cost per case diagnosed.*
3. The ICER of screening for liver cirrhosis in people with T2DM.
4. The number of cancer deaths avoided by screening (as per Markov modelling).
5. The number of patients diagnosed on VCTE with ≥F2 disease (defined as a liver stiffness of ≥8.2 kPa).^21^

*See (i) for the definition of a ‘case’.

### Participants

#### Inclusion criteria

Any adult (≥18 years) patient with a known diagnosis of T2DM according to the primary care record in the Hampshire, Wiltshire, Dorset and the Isle of Wight (all UK) areas will potentially be eligible to participate. Non-English-speaking patients will be eligible for inclusion.

#### Exclusion criteria

- <18 years of age.
- Evaluated for liver disease with either an ELF test or VCTE in the 2 years before the date of consent.
- A known prior clinical diagnosis of significant liver disease (significant fibrosis or cirrhosis and in active hospital follow-up) due to any cause.
- A known diagnosis of autoimmune hepatitis, primary biliary cholangitis and primary sclerosing cholangitis or viral hepatitis (irrespective of whether this has progressed to fibrosis or cirrhosis).

### Setting

The study will be conducted in 16–20 primary care practices and diabetes community care hubs in Wessex (including Hampshire, Wiltshire, the Isle of Wight and Dorset (UK)). The setting of the study is important as it includes a range of existing community liver pathways which means the intervention is compared with a diverse representation of standard care—which is a representative of diverse interpretations of the current NICE guidelines.^32^

Community hubs will be used for research data collection including VCTE and blood sampling. Primary care centres will be identified via the local Primary Care Network and the Primary Care NIHR clinical research network. The number of practices we are using is justified in the later dedicated sections of the form.

Recruitment will take place from January 2024 and will be complete by April 2025. Outcome data collection will be completed by June 2026, and the cost-effective analysis will be completed by the study end date of 1 September 2026.

### Participant identification

Primary care centres will identify potential participants from their patient records. The research team will provide these practices with a search query to run on their patient management systems (SystmOne or EMIS) (see online supplemental material and trial website (reflexstudy.org)). Flagged patients will be screened for eligibility by practice staff. The patients on the list of potential participants will be sent a text advising them about the study, where they can access further information and who to contact if they would like to self-refer their interest in participating (see online supplemental materials).

### Consent and randomisation

If a participant contacts the research team, they will be sent an information sheet and given time to consider participation before providing written consent to the research team (see online supplemental material). After giving consent, each participant will be randomised. To ensure equal numbers of patients within each arm of the study, we will use block randomisation with a block size of 4. Blocks will be used to ensure a balance between the participants in each arm of the study—strata will be sex, age group and alcohol consumption. This will be managed by the Southampton NIHR Biomedical Research Centre team using randomisation software.^39^

### Arm 1—screening

Participants in this arm will be referred by the research team directly for liver fibrosis assessment at a community hub. This assessment will include VCTE and venepuncture for an ELF test and a FIB-4 index. The result of the VCTE and any abnormalities identified in the blood tests will be managed by the local liver disease care pathway (as per the usual care arm described below). VCTE will be performed by an experienced single operator after a minimum of a 3-hour fast and previously published criteria for a valid reading will be applied to each participant.^40^

### Arm 2—standard care—NICE guidelines based T2DM + additional risk factor testing

Participants in the standard care arm will not be contacted for VCTE, and following baseline data collection will have no further contact with the research team during the follow-up period—outcomes will be collected remotely from the medical record (see below).

**Figure 1:**
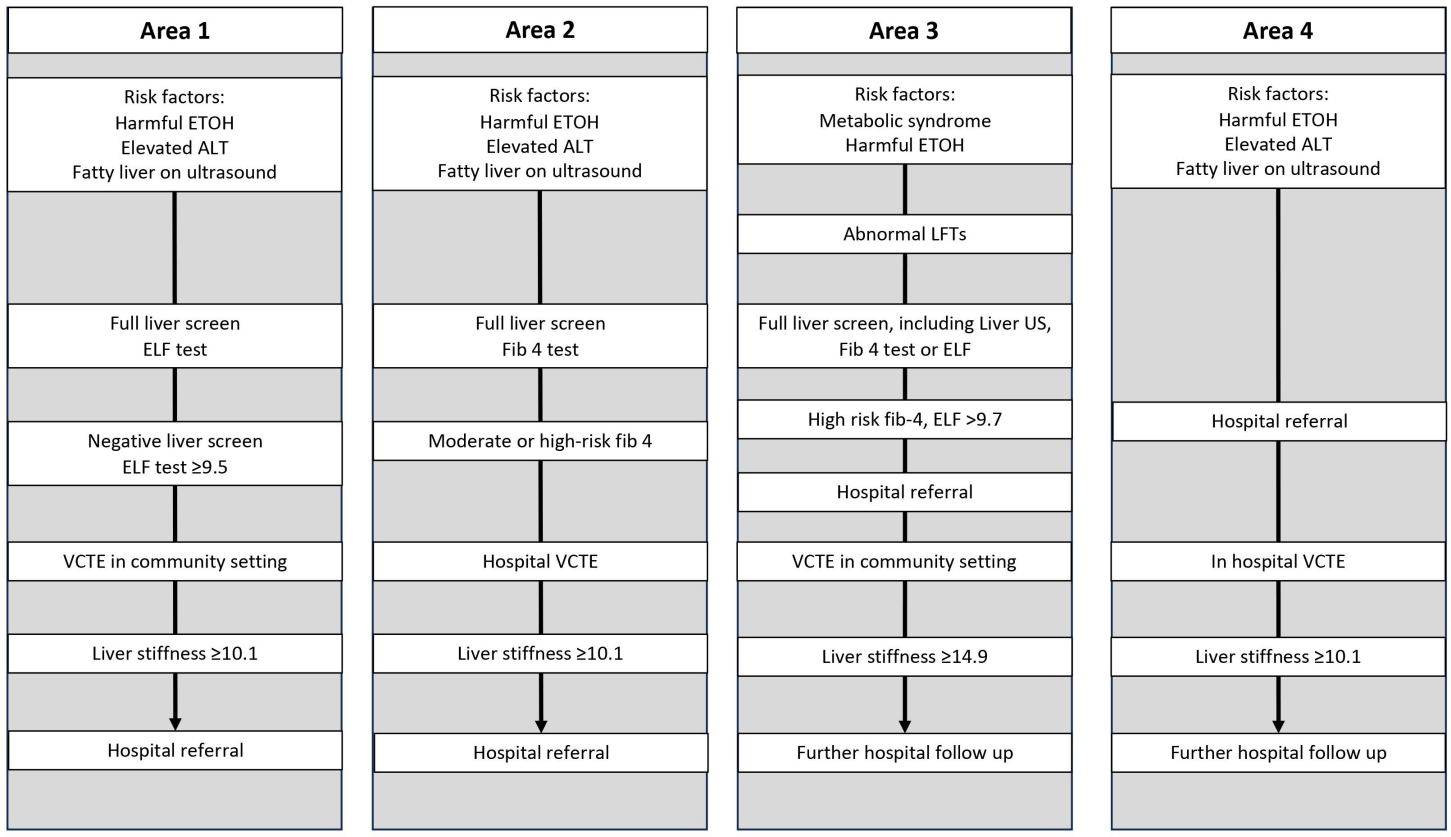
An overview of usual care for liver disease assessment and management within primary and secondary care liver services in study areas—highlighting the complexities and subtle variations in practice. ALT, alanine transaminase; ELF, enhanced liver fibrosis; ETOH, alcohol; FIB4, fibrosis-4; LFT, liver function test; VCTE, vibration-controlled transient elastography.

Standard care varies across the study area but is based on 2016 NICE guidance (NG49) (figure 1).^32^ In the 2016 NICE NAFLD guideline, the presence of T2DM does not trigger an assessment for liver disease in the absence of other specific risk factors.^32^ ‘Risk factors’ to enter standard care vary in the study areas but broadly include harmful alcohol consumption, an elevated ALT and a fatty liver on ultrasound examination. If risk factor thresholds are met, then the usual care pathway varies further, but in all areas involve VCTE with or without a biomarker for liver fibrosis (eg FIB-4 or ELF) (figure 1). The variation in standard care is very important as it increases the external validity of our study by being representative of the heterogeneity across the UK.

After discussion with our patient and public involvement (PPI) groups, participants included in the standard care arm will be given the opportunity to undergo VCTE and a biomarker test to assess them for liver fibrosis >12 months following randomisation (arranged at mutual convenience with the research team).

### Data collection

#### Baseline data collection

All participants will give consent for access to their primary care records. These, alongside a brief questionnaire, will provide participant baseline data including demographics, medication and co-morbidities that cover the Charlson index^41^ (giving an overall score for co-morbidity) and other prevalent co-morbidities in the study population (table 1).^42^ Participants are not asked to complete further data collection activities during the 12-month follow-up period as we want to minimise the potential Hawthorne effect in our control group. We are concerned that prolonged exposure to the research team could lead usual care participants to change their behaviour and either seek or perhaps decline liver assessment.^43^

**Table 1:** Baseline participant characteristics that will be collected and where the data will be collected

Baseline demographic characteristic	Collected at recruitment	Can be collected via EMIS/SystmOne*
Age, years	✓	
Sex, male (%)	✓	
Ethnicity (white European or minority ethnic group)	✓	
Alcohol consumption (AUDIT-C score)	✓	
Measured height (cm)		
Measured weight (kg)	✓	
Smoking status (current, ex, never)		✓
Index of multiple deprivation (from postcode)	✓	
Duration of diabetes (years)		✓
Medical treatment for diabetes—tablets or insulin (currently, previously, never)	✓	
**Currently prescribed medications**		
Antiglycaemic treatment (any)	✓	
Sulphonylurea (eg, gliclazide)	✓	
Metformin	✓	
Insulin	✓	
GLP-1 agonist (eg semaglutide)	✓	
Pioglitazone	✓	
SGLT2 inhibitor (eg flozins)	✓	
Anticoagulants (DOAC or warfarin)	✓	
Antihypertensives (any)	✓	
ACE (eg ramipril)	✓	
ARBs (eg candesartan)	✓	
B-blockers (eg bisoprolol)	✓	
Thiazides (eg BTZ)	✓	
Calcium channel blockers (eg amlodipine)	✓	
Antidepressants	✓	
Fibrates	✓	
Statins	✓	
**Co-morbidities (to calculate Charlson co-morbidity index)**		
Definitive or probable previous myocardial infarction	✓	✓
Congestive heart failure (dyspnoea with response to CHF medication)	✓	✓
Peripheral vascular disease (intermittent claudication, previous by-pass grafting)	✓	✓
Any end organ damage due to T2DM	✓	✓
Moderate to severe chronic kidney disease	✓	✓
Solid tumour (non-localised, metastatic)	✓	✓
Lymphoma (either cured, in remission or active)	✓	✓
Hemiplegia	✓	✓
AIDs	✓	✓
Peptic ulcer disease	✓	✓
Connective tissue disease (eg SLE, rheumatoid arthritis, not osteoarthritis)	✓	✓
**Additional prevalent comorbidities in patients with T2DM**	✓	✓
Hypertension	✓	✓
Asthma	✓	✓
Hypothyroidism	✓	✓

* EMIS and Systm1 are primary care software programmes used throughout England.

ACEangiotensin converting enzymeARBangiotensin receptor blockerAUDIT-CAlcohol Use Disorders Identification Test-ConsumptionBTZbendroflumethiazideCHFcongestive heart failureDOACDirect Oral AnticoagulantsGLP-1glucagon-Like peptide-1 receptor agonistSGLT2sodium/glucose cotransporter 2SLESystemic Lupus ErythematosusT2DMtype 2 diabetes mellitus

#### Primary outcome data collection

The primary outcome—referral to HCC surveillance following a referral with suspected liver disease from primary care will be assessed by the research team from each participant’s healthcare records. Participants will not need to be recontacted for outcome data. For standard care participants, the primary care record will be reviewed for a referral letter to secondary care or a community liver assessment service that was sent within 12 months of randomisation. For both trial arms, records will be reviewed for evidence (eg a letter from hepatology services) that the patient has been enrolled in HCC surveillance. The primary care record review will take place up to 30 months from randomisation to ensure enough time for definitive decisions regarding HCC surveillance to have been made by the clinical team.

#### Cost data collection

We will collect micro-costs^44^ on the following components of the pathway:

- Item costs for ELF and FIB-4 tests and venepuncture costs.
- Nursing time for: venepuncture, VCTE, results delivery and onward referral.
- Cost per VCTE assessment including equipment, equipment servicing and training.
- Community venue hire for liver assessment.

### Data management plan

Participant data will be managed according to the study data management plan which is available on the study website (reflexstudy.org). Study data, including participant-identifiable data will be stored securely by ethical approvals.

### Data analysis

#### Primary outcome

We will conduct an ‘intention to diagnose’ analysis for the primary outcome where all participants undergoing randomisation will be analysed within the group to which they were assigned, regardless of whether they engaged with the diagnostic process following referral within their study arm. Logistic regression will be used to compare the binary outcome between the standard care and intervention arms. Exact or penalised likelihood estimation methods will be used to avoid the small-sample bias that otherwise would be present with such small, expected outcome numbers. Loss to follow-up (LTFU) and missing data will be managed by our LTFU management plan (see online supplemental material).

#### Cost-effectiveness analysis

For the cost-effectiveness evaluation, data from the study will be incorporated into a decision analytical model (developed in Microsoft Excel). These data include: the micro-costs of testing and follow-up, drop-out rates from the diagnostic pathways (usual care and screening), the relative proportions of different stages of liver disease and the demographic characteristics of the cohorts.

**Figure 2:**
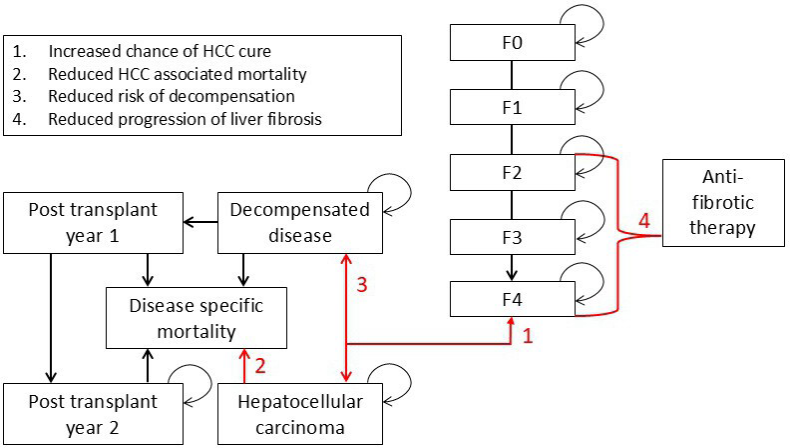
Markov model structure is used to calculate the incremental cost-effectiveness of different testing strategies. The findings from the trial will parameterise this model. Numbers 1–4 correspond to the benefits of early detection that will be incorporated into the modelling. HCC, hepatocellular carcinoma.

The model will consist of a decision tree for the diagnostic process and a Markov state transition model for the long-term disease process (figure 2). It will estimate the quality-adjusted life years (QALYs) and costs associated with liver disease. The model structure will be similar to previous models for HCC surveillance (eg,^34 36^) and calculate the difference in costs and QALYs between different testing approaches and no testing. Patients with characteristics based on our study population and study outcomes will enter the model. The model will have 1 year cycles and a lifetime horizon (ie until the cohort age is 100 years). Costs will be calculated using an NHS and Personal Social Services perspective. Costs and utilities for the model health states will be taken from a targeted review of the medical literature.

Our base-case analysis will closely match real-world practice. In both cohorts, patients identified with liver cirrhosis and referred for HCC surveillance will enter a separate health state—named F4_SURV. Based on recently published data, participants in this health state who develop HCC will have a higher chance of cure (ie return to their original F4 SURV health state) and a lower chance of progression to death or transplantation.^16^ Similarly, a proportion of those in F4_SURV will have a lower risk of progressing to a decompensated state that reflects the real-world number of participants who commence B blockers by recent guidelines.^45 46^ Participants identified with F2 or F3 disease will enter monitoring states (F2_Mon and F3_Mon) and undergo biannual assessment for progression to F4 disease. Monitoring will stop when participants in the model reach 80 years of age.

As part of our base case analysis, we will calculate the cost-effectiveness (cost per QALY) of four testing strategies that are broadly reflective of current testing strategies in the study region and the NHSE pilot (described in the background). These will be compared against ‘no testing’ and presented as ICERs that can then be compared between strategies.

1. Usual care.
2. Reflex testing with VCTE only (ie everyone offered VCTE).
3. FIB-4 then VCTE for patients with FIB-4>3.25.
4. ELF then VCTE for patients with an ELF >9.5.

We will conduct probabilistic sensitivity analyses where model parameters are probabilistically varied across pre-specified distributions and ranges. The results of the probabilistic sensitivity analyses will be presented as a scatter plot and a cost-effectiveness acceptability curve.

**Figure 3:**
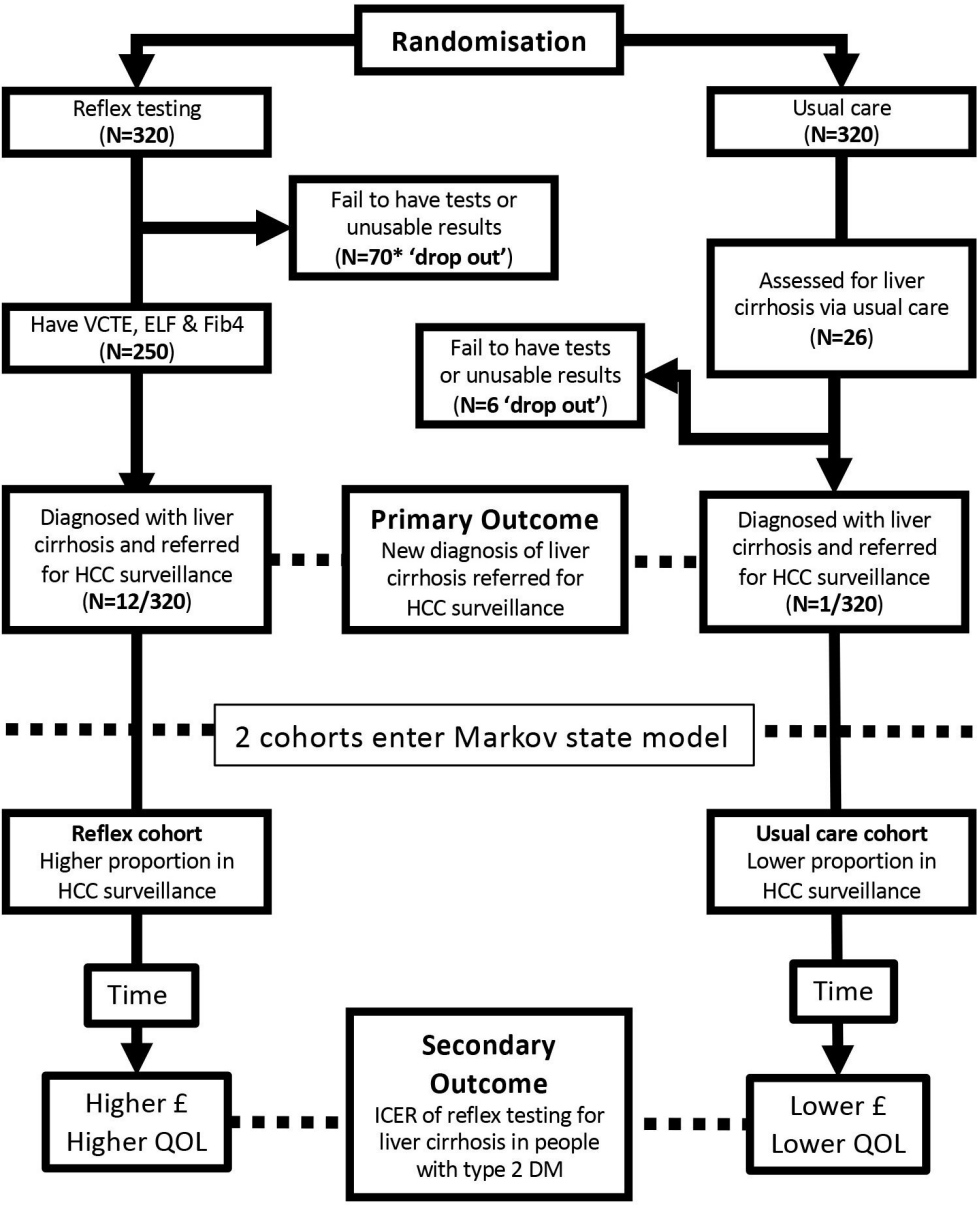
Study flow chart showing how the study arms and nested cost-effectiveness evaluation. The rationale for the study sample size is also conveyed. DM, diabetes mellitus; ELF, enhanced liver fibrosis; FIB4, fibrosis-4; HCC, hepatocellular carcinoma; ICER, incremental cost-effectiveness ratio; QOL, quality of life; VCTE, vibration-controlled transient elastography.

Finally, we will conduct a one-way sensitivity analysis varying the input parameters in the model and scenarios around the main model assumptions. Specifically, we will test a scenario where we introduce a hypothetical anti-fibrotic agent that is given to patients in the F2_Mon and F3_Mon health states. As part of this, we will conduct a threshold analysis where we will calculate ICERs for the hypothetical drug at different levels of therapeutic effectiveness. Anti-fibrotic therapy is not part of our base-case analysis as it is not currently part of usual care in England. Figure 3 shows a study flow chart showing how the study arms and nested cost-effectiveness evaluation are linked. The rationale for the study sample size is also conveyed.

### Sample size

We will aim to recruit 320 patients into each arm of this study—640 patients in total (figure 3). A sample of this size will enable us to address the primary outcome, with a minimum power of 80% after allowing for a very conservative 25% drop-out rate from the diagnostic pathway in both arms. A more realistic drop-out rate would be 5% which would give the power to test the primary outcome of >90%.

We are concerned that the conduct of our study may increase liver disease diagnosed via usual care due to the Hawthorne effect on participants randomised to usual care or on primary care physicians who are more likely to request testing because they are, as a consequence of participation, more aware of liver disease.^43^ Our sample size therefore also accounts for a doubling of background liver fibrosis testing in usual care. The background testing activity for liver disease in the study setting has been very important in calculating our sample size. We have estimated the background testing activity from what we know about the number of patients tested for liver fibrosis who have T2DM in a year and the total population of people with T2DM (figure 3).

All sample size calculations were conducted using nQuery Advisor 7.0.

### Patient and public involvement

To design the trial, we have worked with two PPI representatives (one as PPI lead) and two PPI groups. Our PPI group was struck by the risk of liver cancer in people with diabetes. This was not something they were previously aware of. Both groups of contributors shared the views that cancer and specifically surveillance for liver cancer should be the focus of our research. Our groups are diverse—eight participants in total; two female; two non-white British; and one born in Eastern Europe. The PPI groups have helped develop our study recruitment strategy and our participant-facing study materials. Both groups raised some concerns about the use of a control arm. They advised us to ensure liver assessment was offered to all participants at the end of the study, and this has been incorporated into our study procedures.

## Discussion

The application, effectiveness and cost-effectiveness of screening for liver disease in patients with T2DM have not been well studied. Despite this, it is now recommended practice in some countries and subject to national clinical pilots in others. We aim to fill this knowledge gap.

The robust assessment via randomised controlled trial of a screening intervention for liver disease in T2DM with an objective primary outcome that is assessed independently of the researchers will have a significant impact. If effective, the trial would provide evidence to justify widespread screening in an enormous and growing proportion of the global population with a reduction in liver death. If not effective, it could prevent further rollout of a massive, costly programme of work that will have significant resource implications for health service systems. Looking forward, the trial will also quantify the effect size required and suitable pricing for novel anti-fibrotic therapies to meet cost-effectiveness thresholds.

A strength of the study design is the incorporation of a usual care arm that is a diverse representation of standard practice where testing for liver disease is applied to a few, selected patients with T2DM. The design therefore allows for real-world comparisons between the status quo and (via the intervention arm) a close representation of what a screening programme for liver disease in patients with T2DM might look like.

## Ethical approval and dissemination plans

The University of Southampton is the study sponsor, ERGO II submission ID 80205. Ethical approval was granted by the West of Scotland Research Ethics Service on 2 August 2023, REC reference 23/WS/0102. Any amendments to the study protocol will require authorisation from the ethical approvers. We expect that participants will be identified with liver disease as part of this study. We will work closely with clinicians in the study areas to ensure they are referred and reviewed in line with local practice. We also have academic clinicians within the study team (RMB and CB) who can support participants if the need arises.

Our PPI group will explore the use of the internet, social media and involvement of community venues (eg mosques, churches, gurdwaras and community centres) to reach marginalised populations and convey the study findings. Our PPI lead will aim to publish articles in local newspapers and newsletters and explore possibilities for translation. We aim to submit our findings in abstract form to the European Liver Conference in January 2026 and submit them to a high-impact liver medicine journal later that year.

## supplementary material

10.1136/bmjopen-2024-088043 online supplemental file 1
