# Supplementary material for: Screening to identify people with type 2 diabetes at risk of liver cancer in primary care: a randomised controlled trial protocol
Source: BMJ Open. 2025 Mar 6;15(3):e088043. doi: 10.1136/bmjopen-2024-088043 (PMC11887308; doi:10.1136/bmjopen-2024-088043)
Supplement: online supplemental file 1 [file bmjopen-15-3-s001.docx]

# REFLEX Supplementary Information

[Supplementary 1 – Search Queries 2](#_Toc184203612)

[Supplementary 2 – EOI 3](#_Toc184203613)

[Supplementary 3 – Introductory Letter 4](#_Toc184203614)

[Supplementary 4 – Eligibility Questionnaire 5](#_Toc184203615)

[Supplementary 5 – Consent Form 6](#_Toc184203616)

[Supplementary 6 – Participant Initial Questionnaire 7](#_Toc184203617)

[Supplementary 7 – PIS 9](#_Toc184203618)

[Supplementary 8 – Poster 13](#_Toc184203619)

[Supplementary 9 – Summary PIS 14](#_Toc184203620)

[Supplementary 10 – TV Feed 15](#_Toc184203621)

[Supplementary 11 – GP Website 18](#_Toc184203622)

[Supplementary 12 – Flyer 19](#_Toc184203623)

[Supplementary 13 – Patient Letter 20](#_Toc184203624)

[Supplementary 14 – Missing data plan 22](#_Toc184203625)

## Supplementary 1 – Search Queries

<https://www.reflexstudy.org/gp-system-queries/>


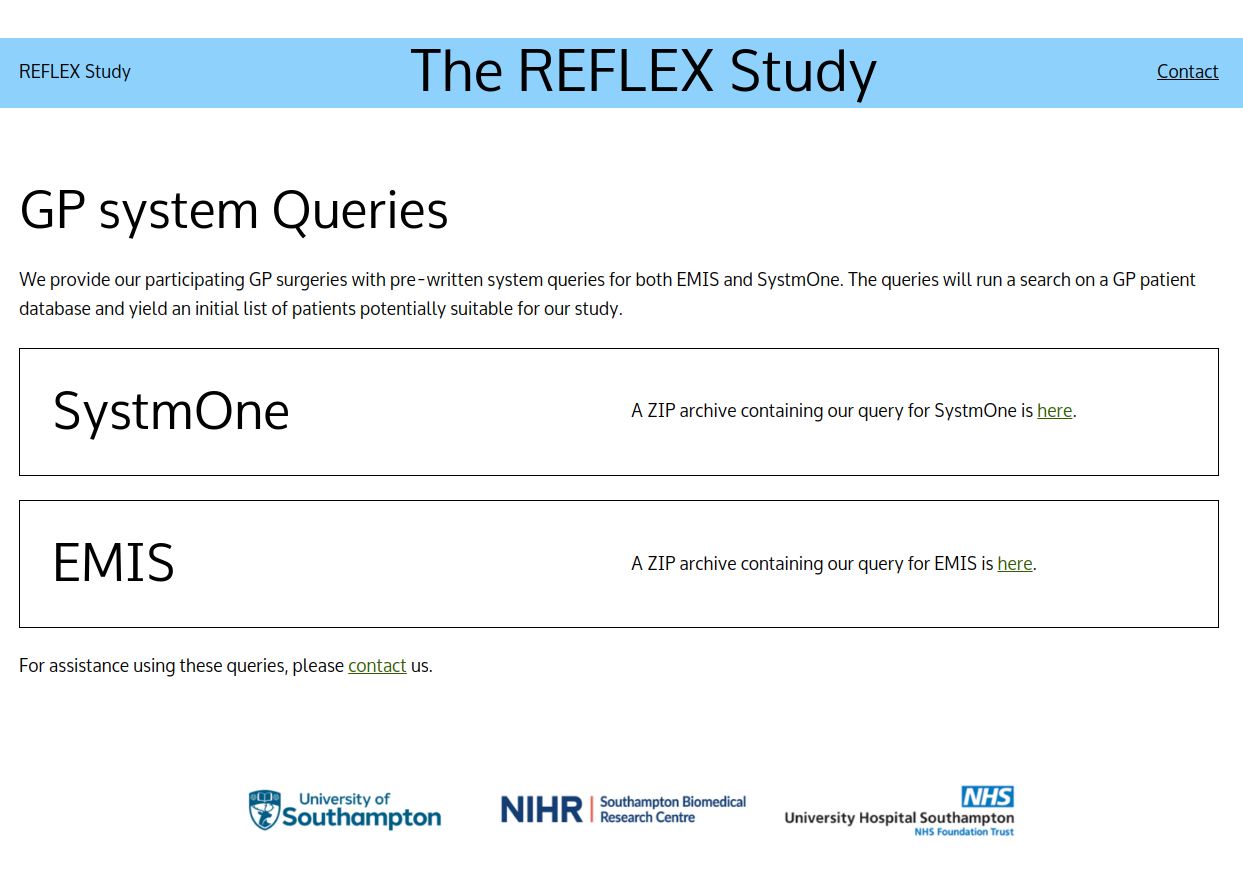


## Supplementary 2 – EOI

<https://www.reflexstudy.org/wp-content/uploads/2023/08/eoi.pdf>


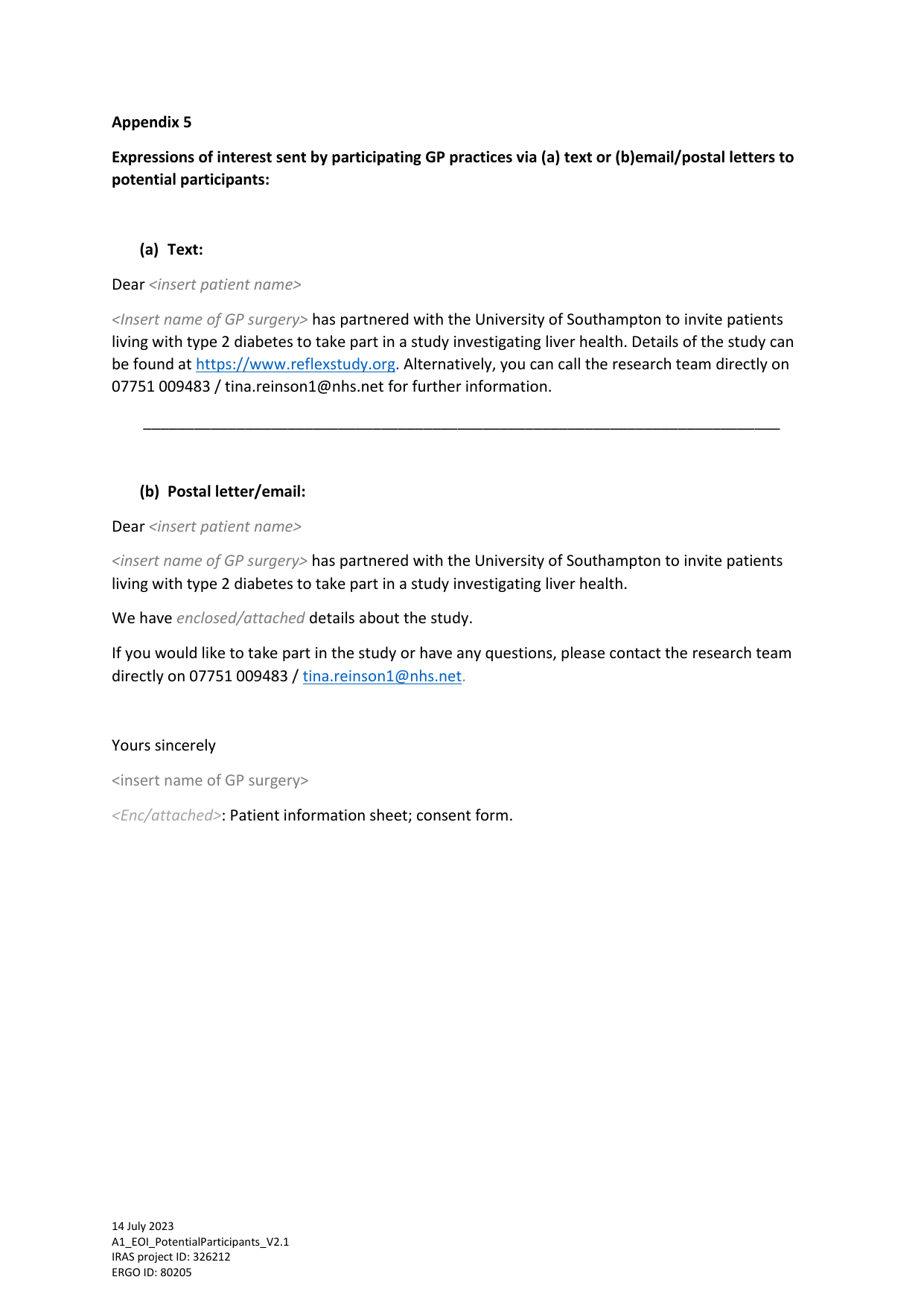


## Supplementary 3 – Introductory Letter

<https://www.reflexstudy.org/wp-content/uploads/2023/08/intro_letter.pdf>


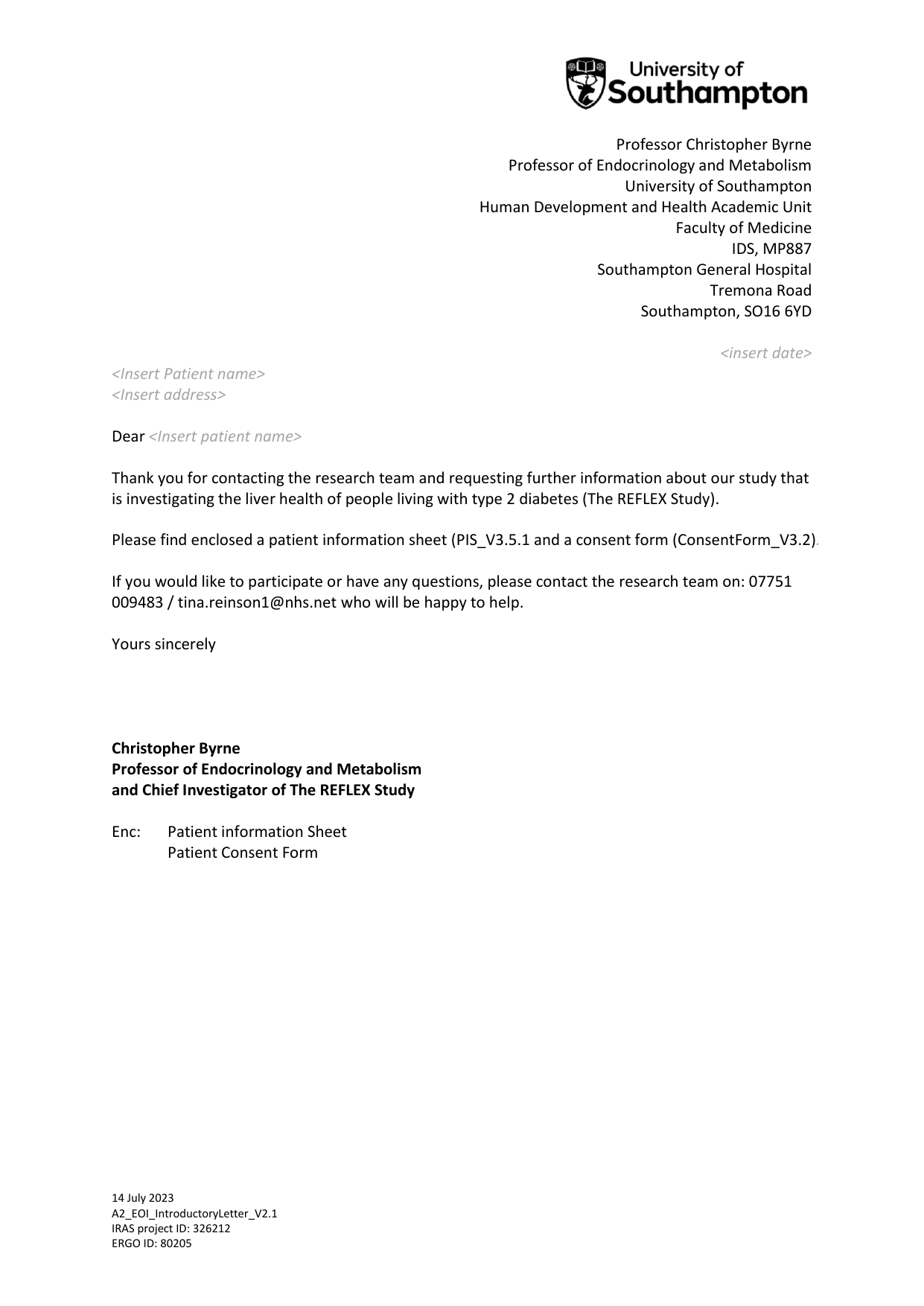


## Supplementary 4 – Eligibility Questionnaire

<https://www.reflexstudy.org/wp-content/uploads/2023/08/eligibility_qs.pdf>


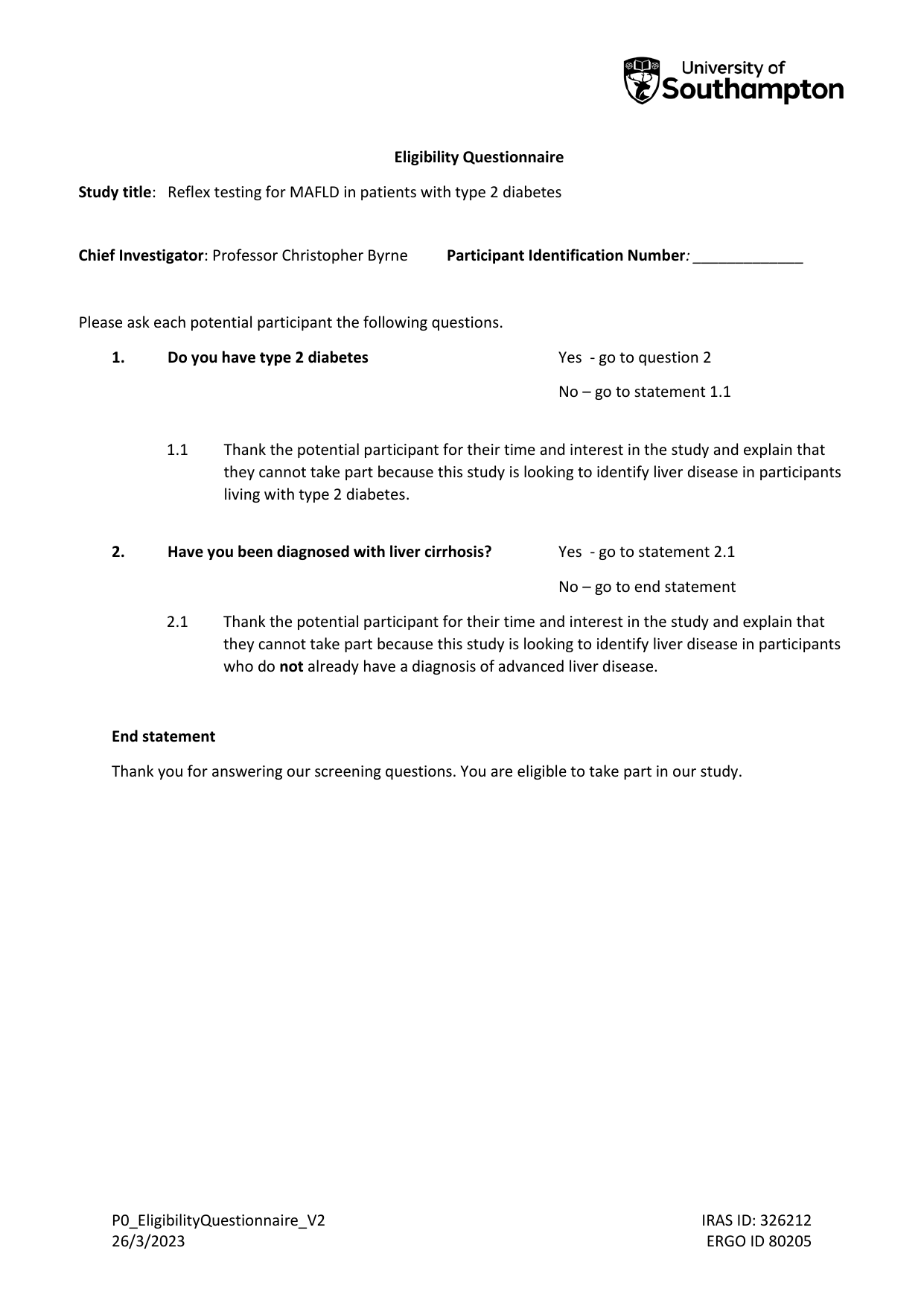


## Supplementary 5 – Consent Form

<https://www.reflexstudy.org/wp-content/uploads/2023/08/consent_form.pdf>


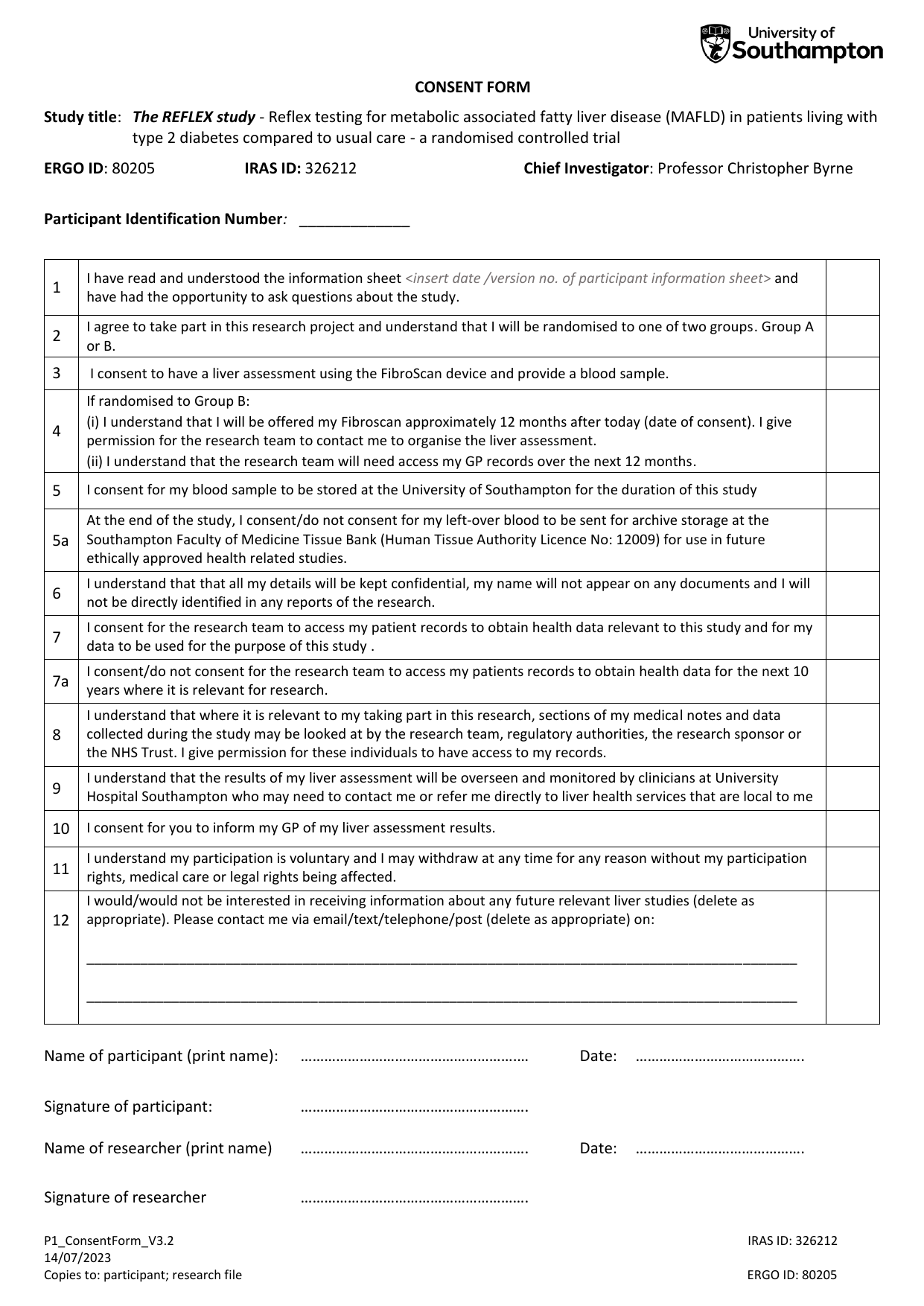


## Supplementary 6 – Participant Initial Questionnaire

<https://www.reflexstudy.org/wp-content/uploads/2023/08/initial_q.pdf>


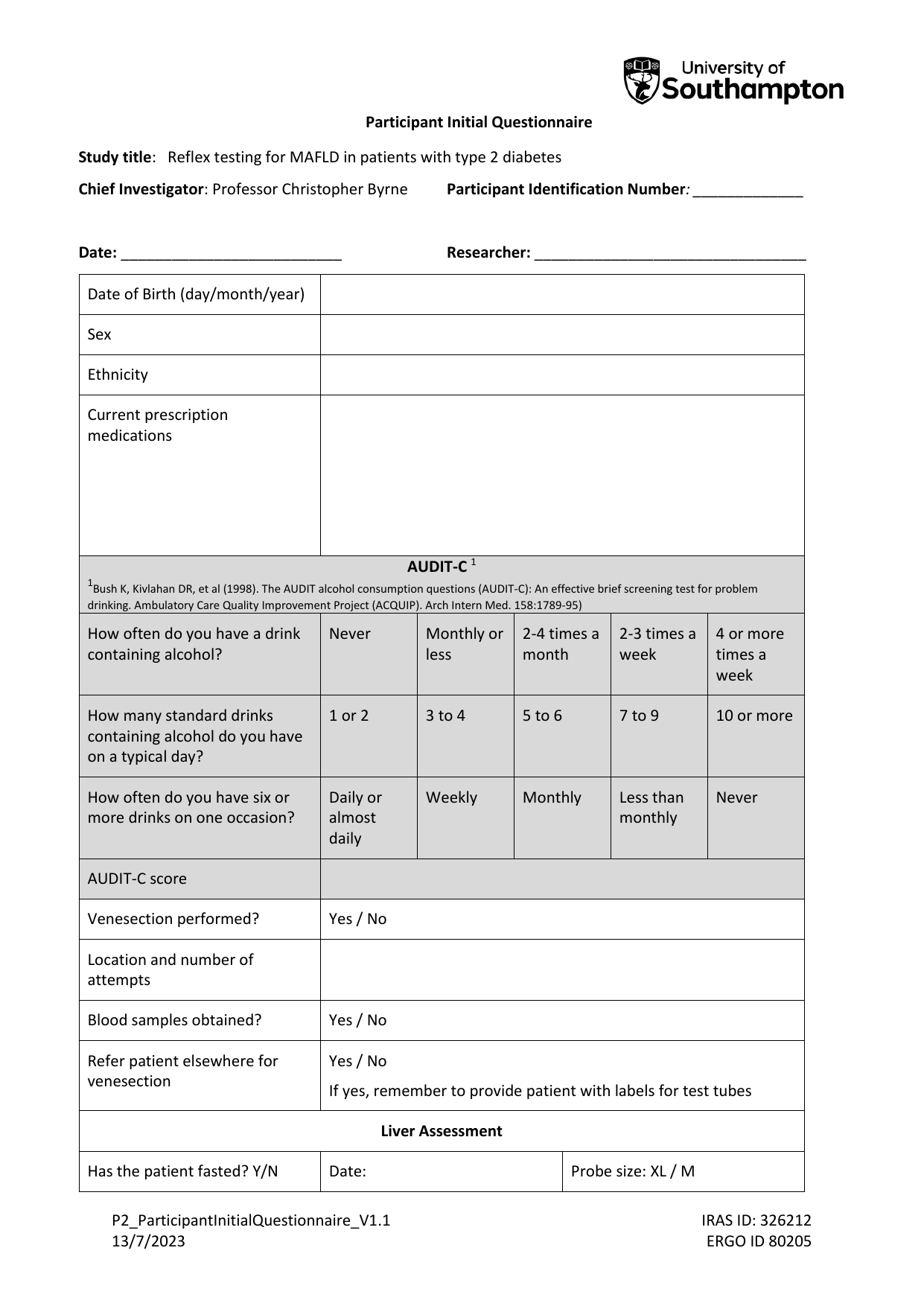


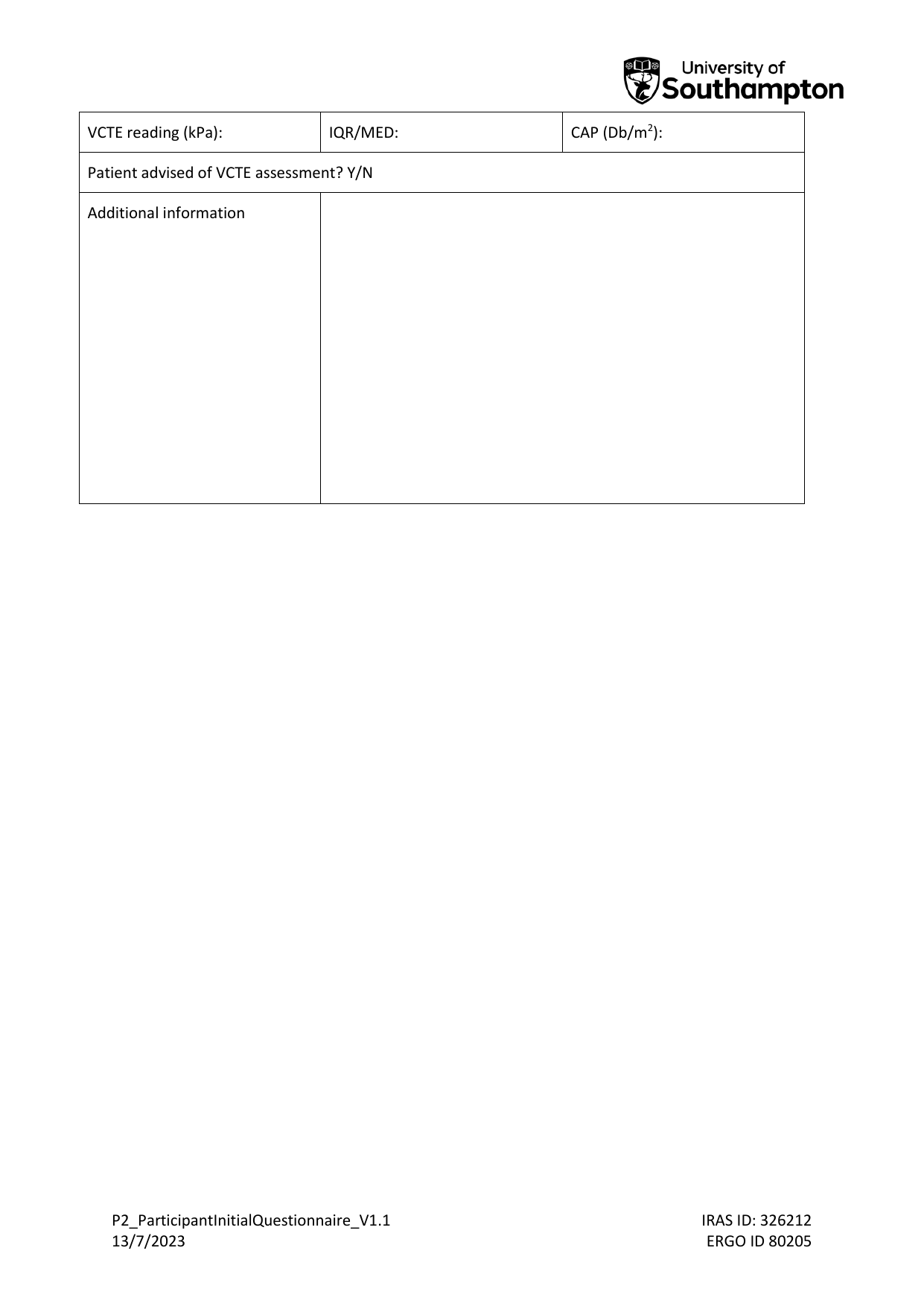


## Supplementary 7 – PIS

<https://www.reflexstudy.org/wp-content/uploads/2023/08/pis.pdf>


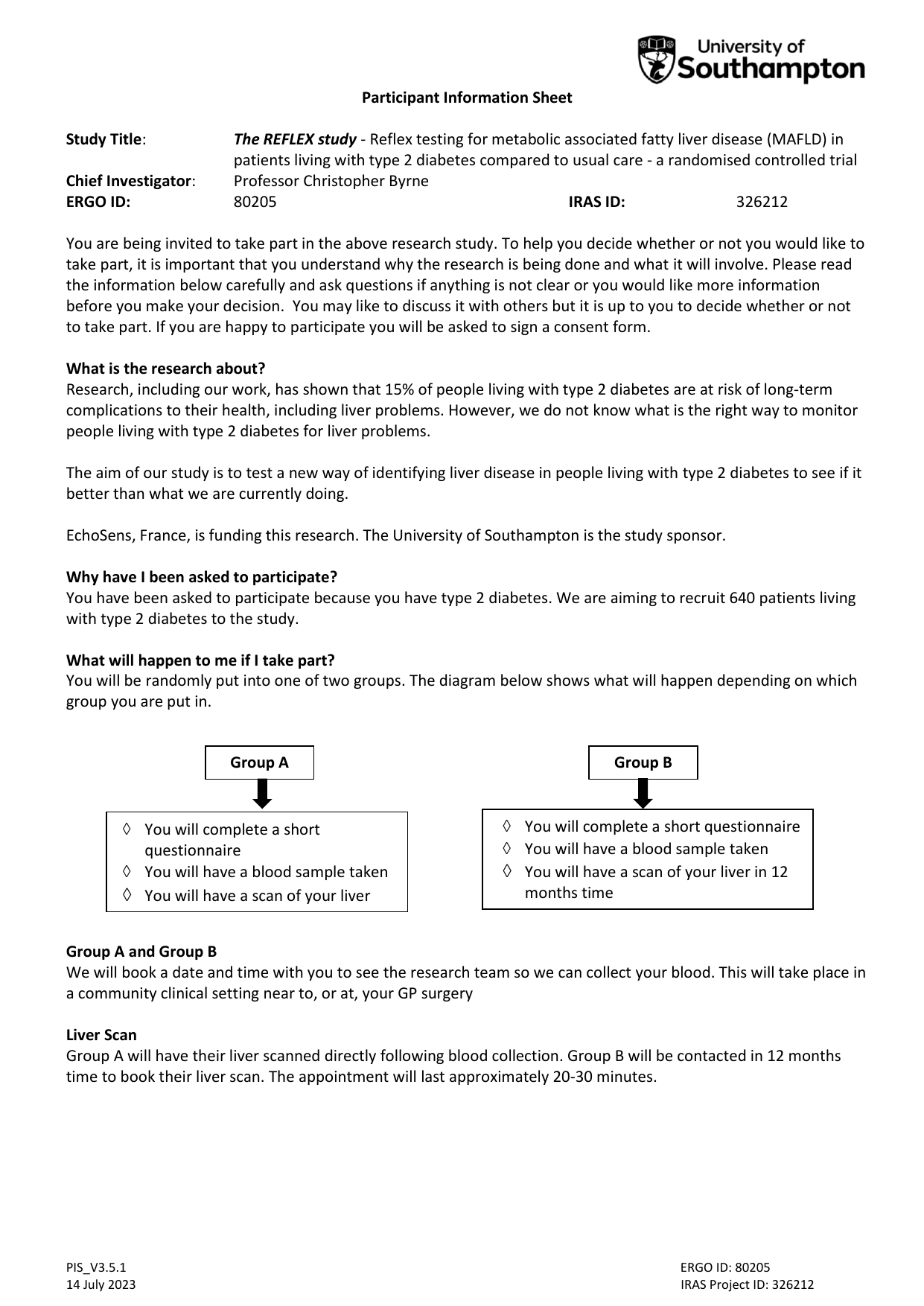


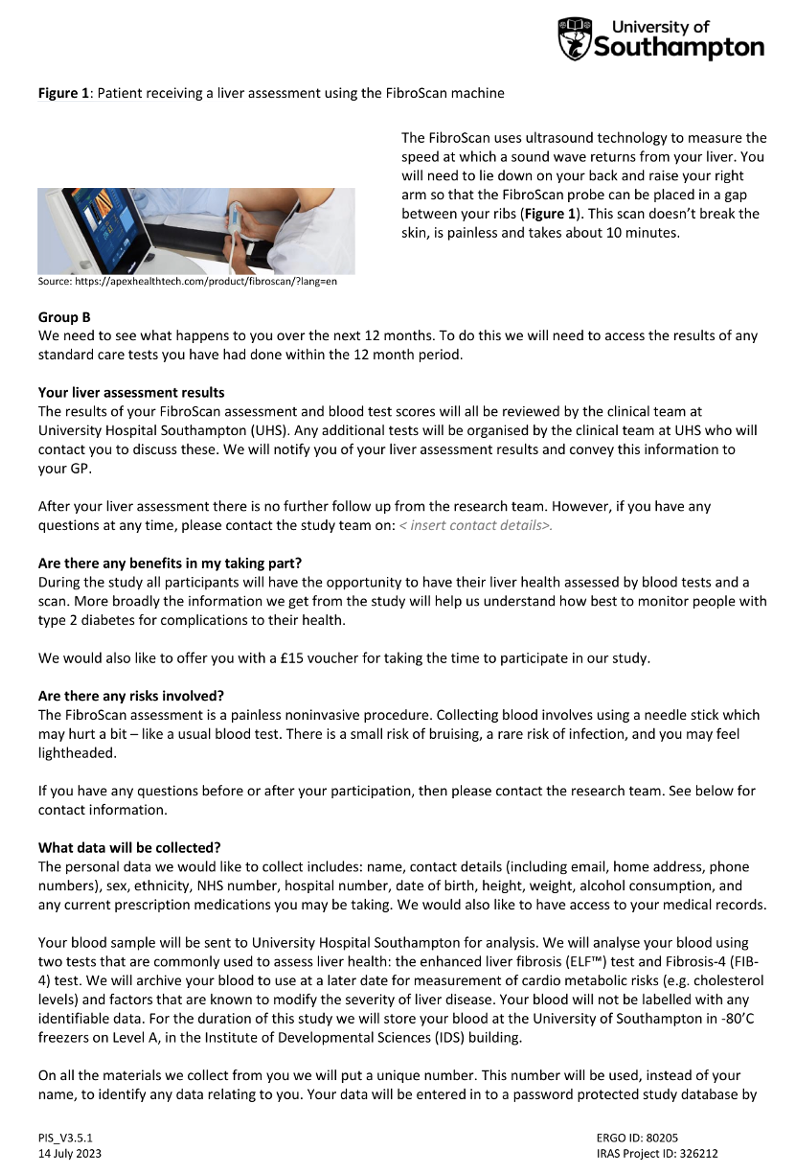


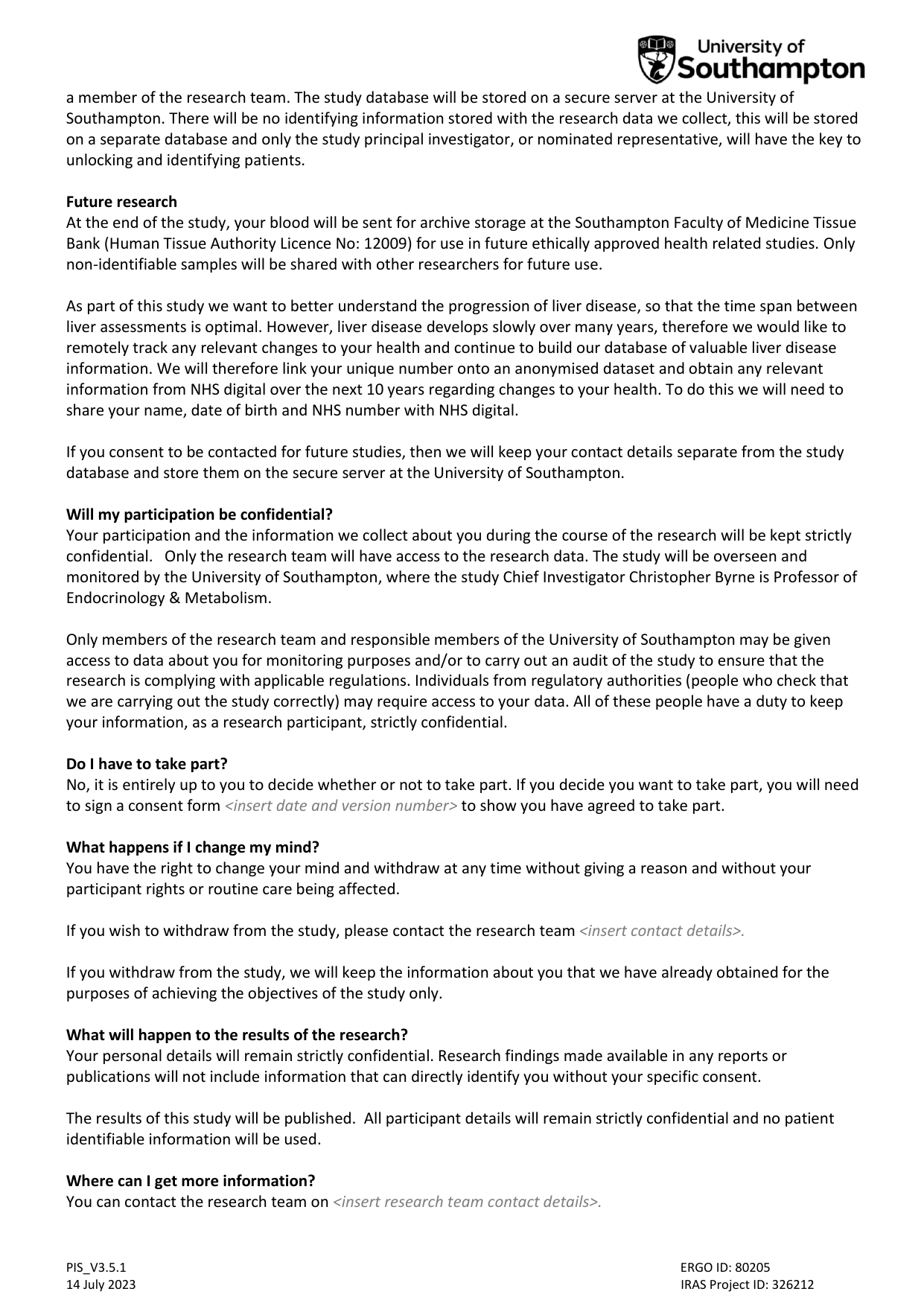


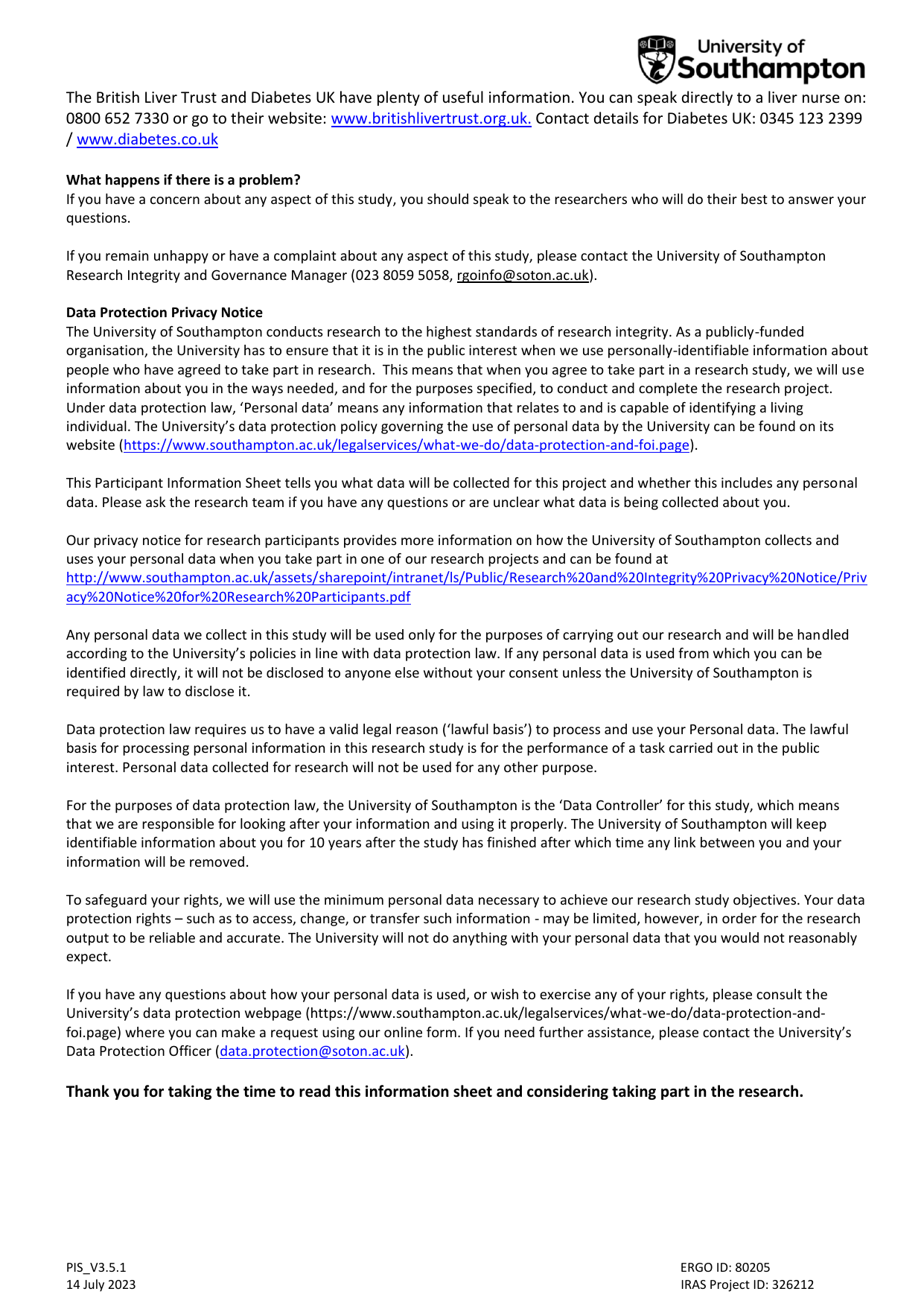


## Supplementary 8 – Poster

<https://www.reflexstudy.org/wp-content/uploads/2023/08/poster.pdf>


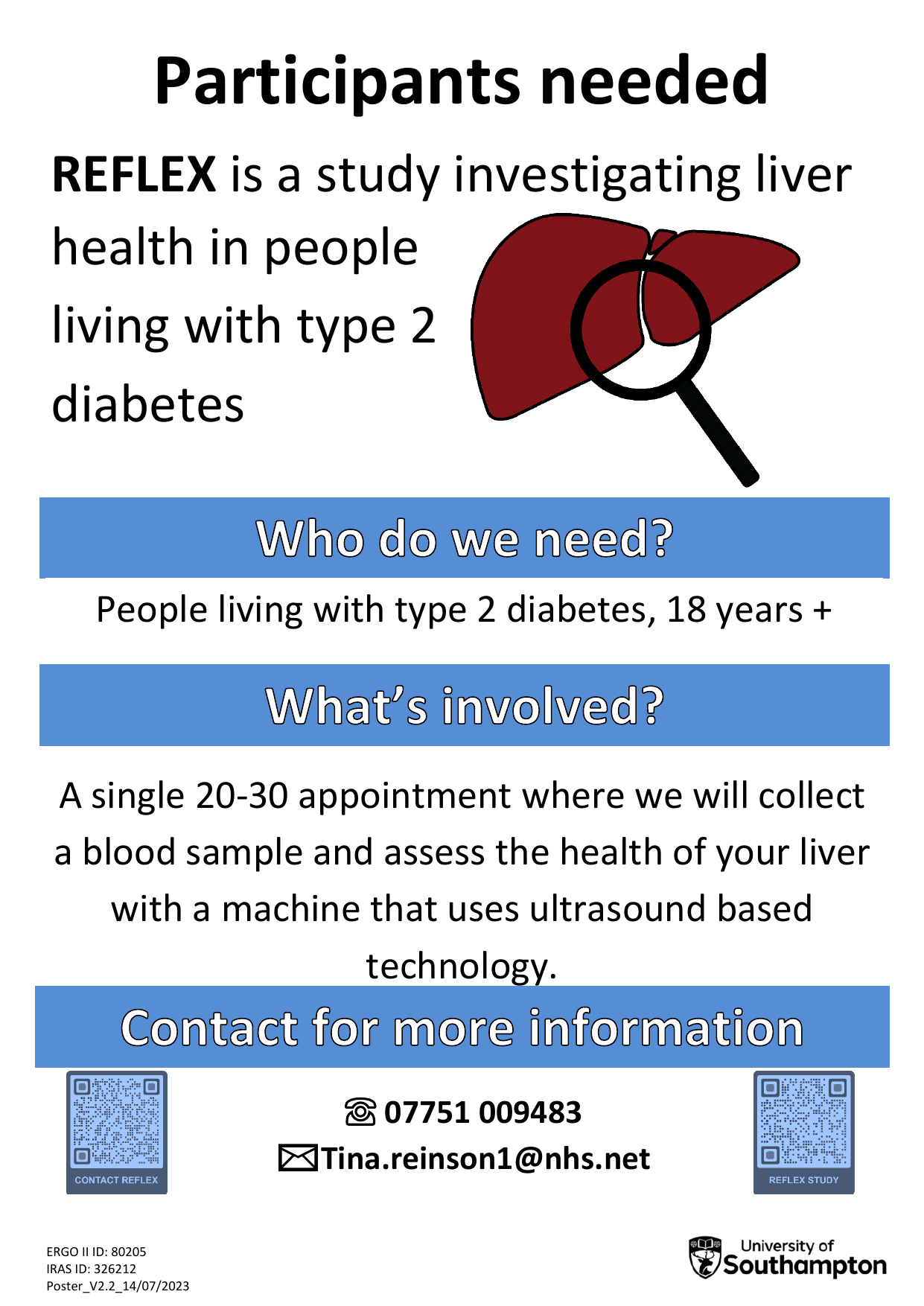


## Supplementary 9 – Summary PIS

<https://www.reflexstudy.org/wp-content/uploads/2023/08/summary_pis.pdf>


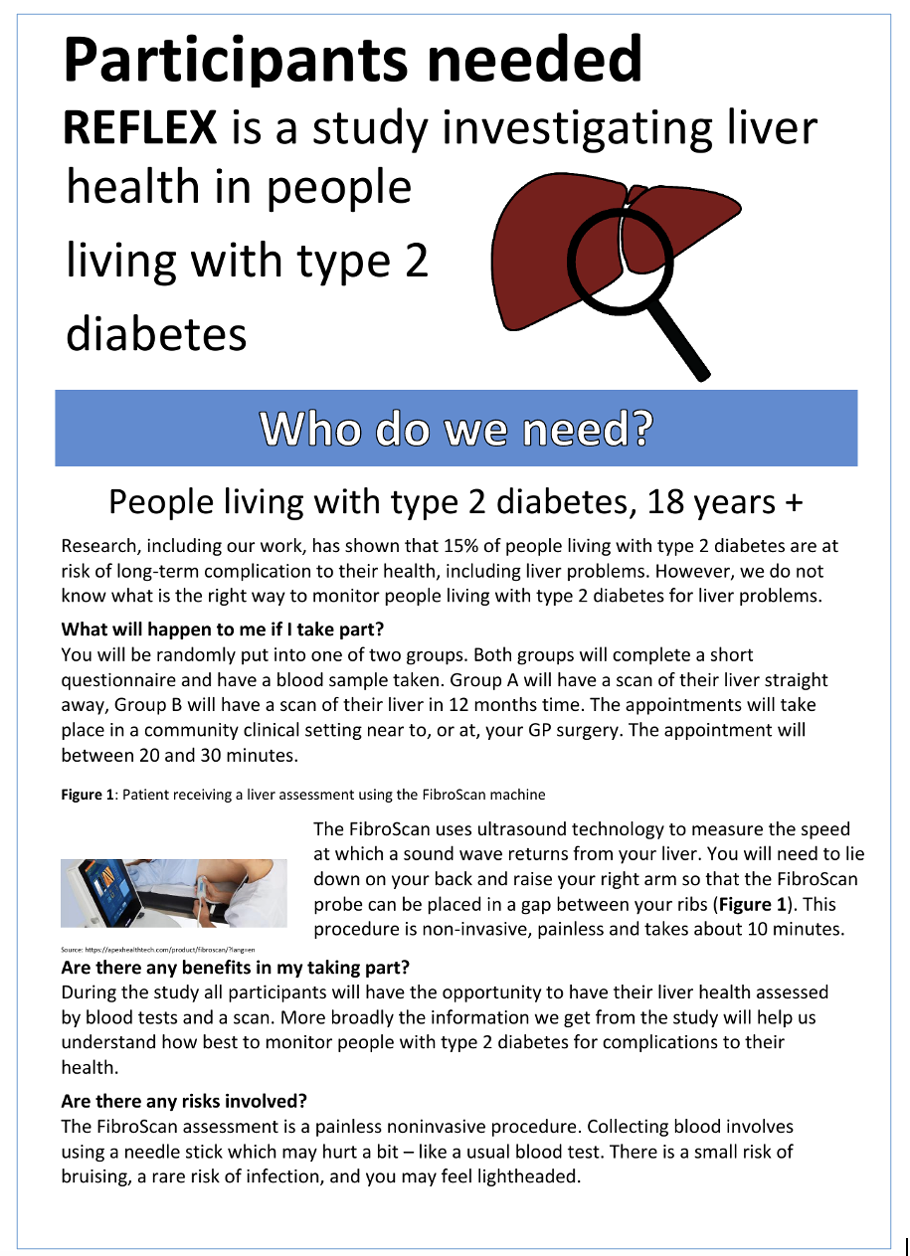


## Supplementary 10 – TV Feed

<https://www.reflexstudy.org/wp-content/uploads/2023/08/TV_feed.pdf>
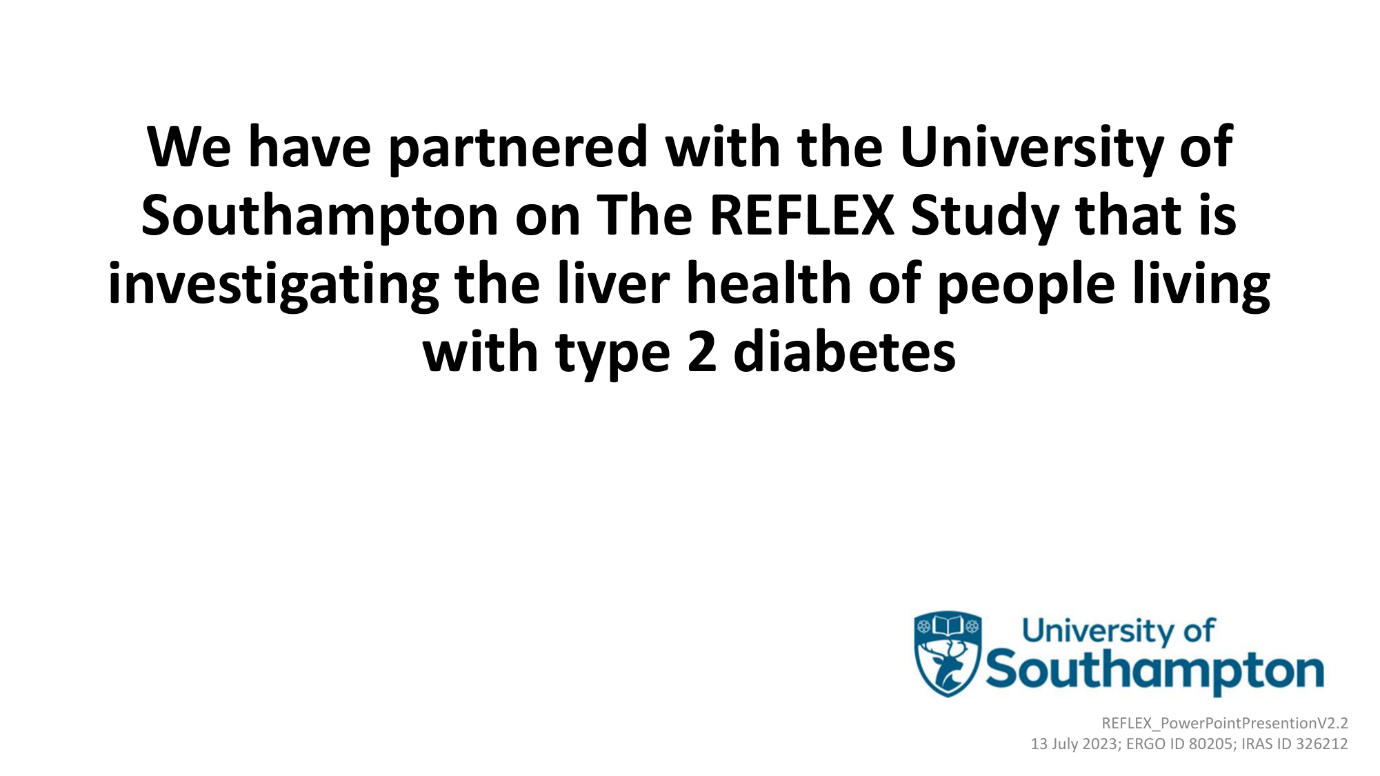

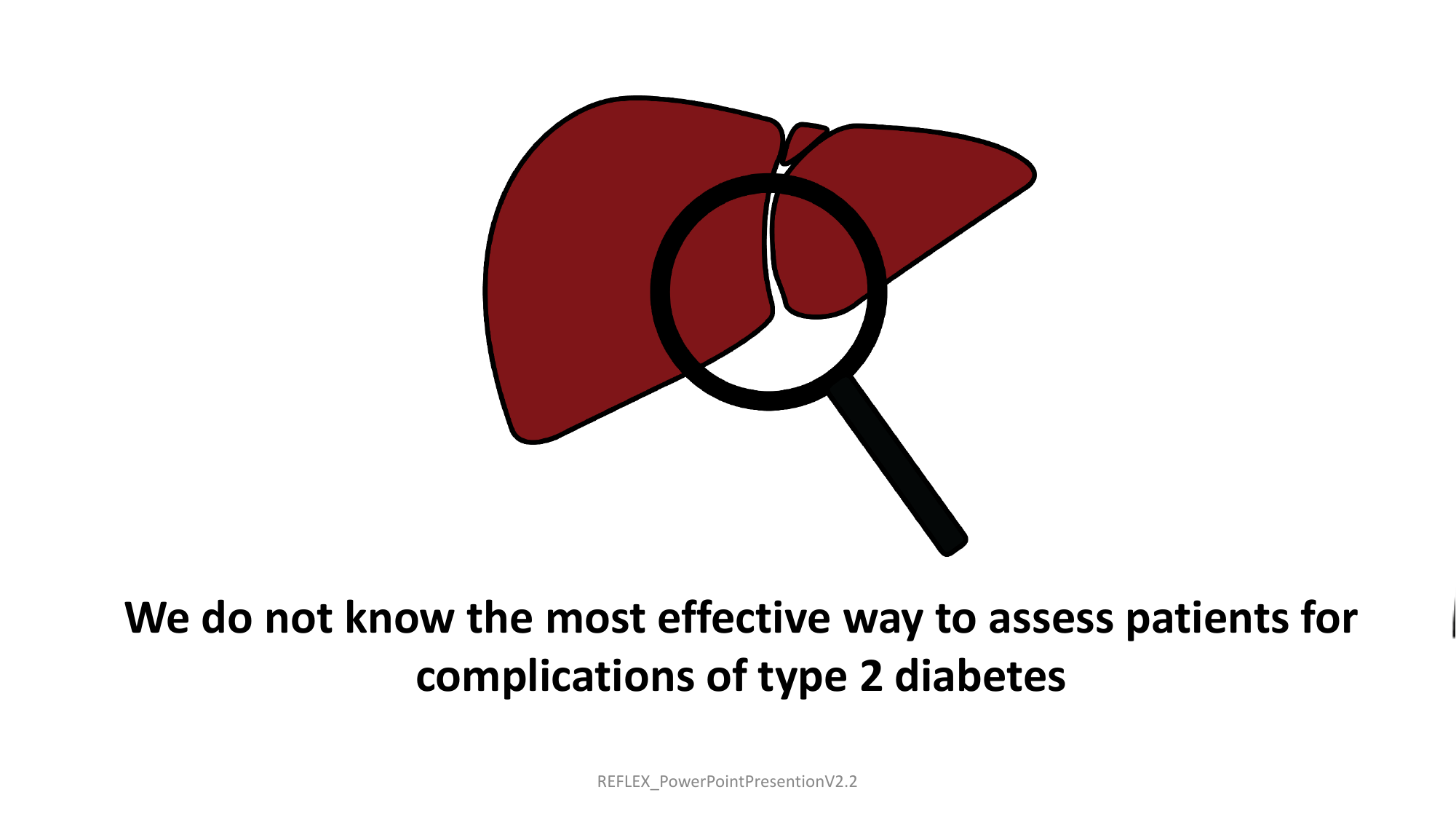

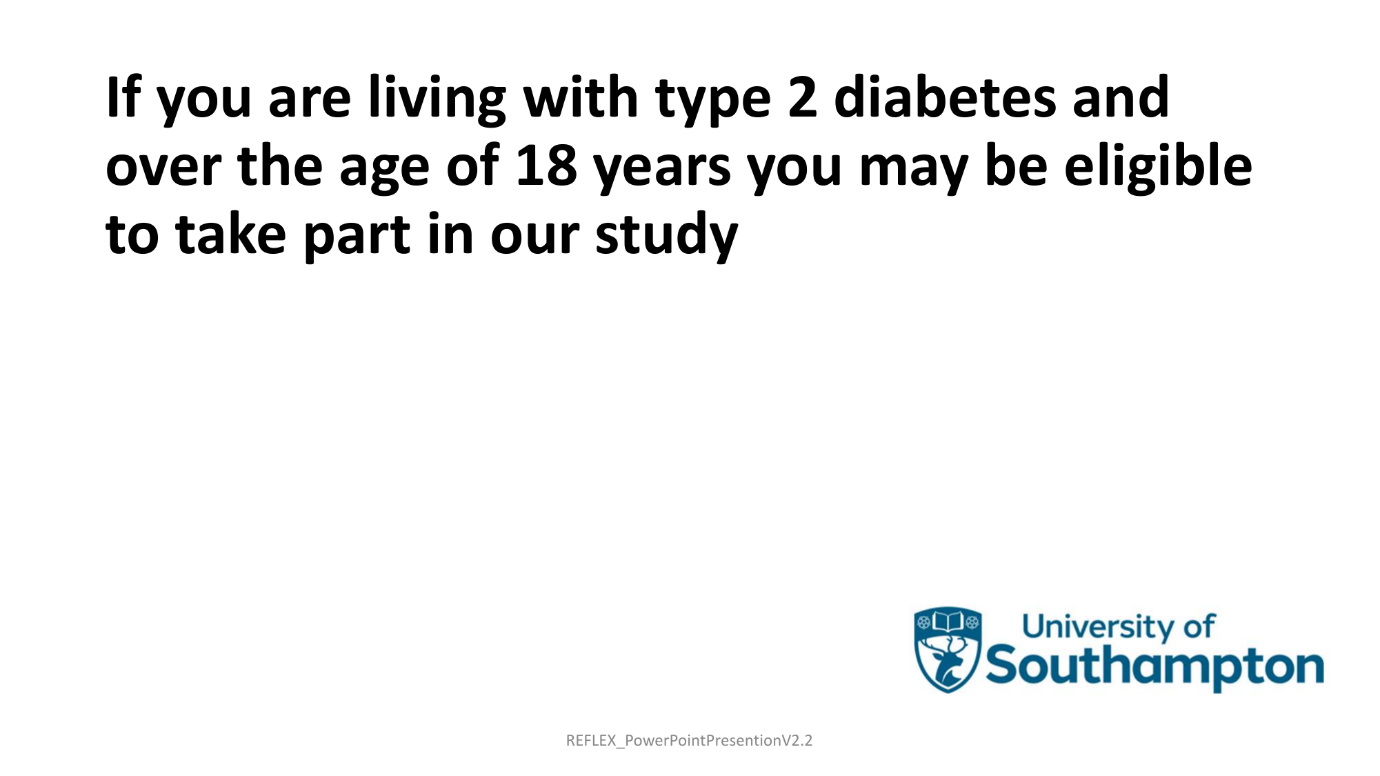

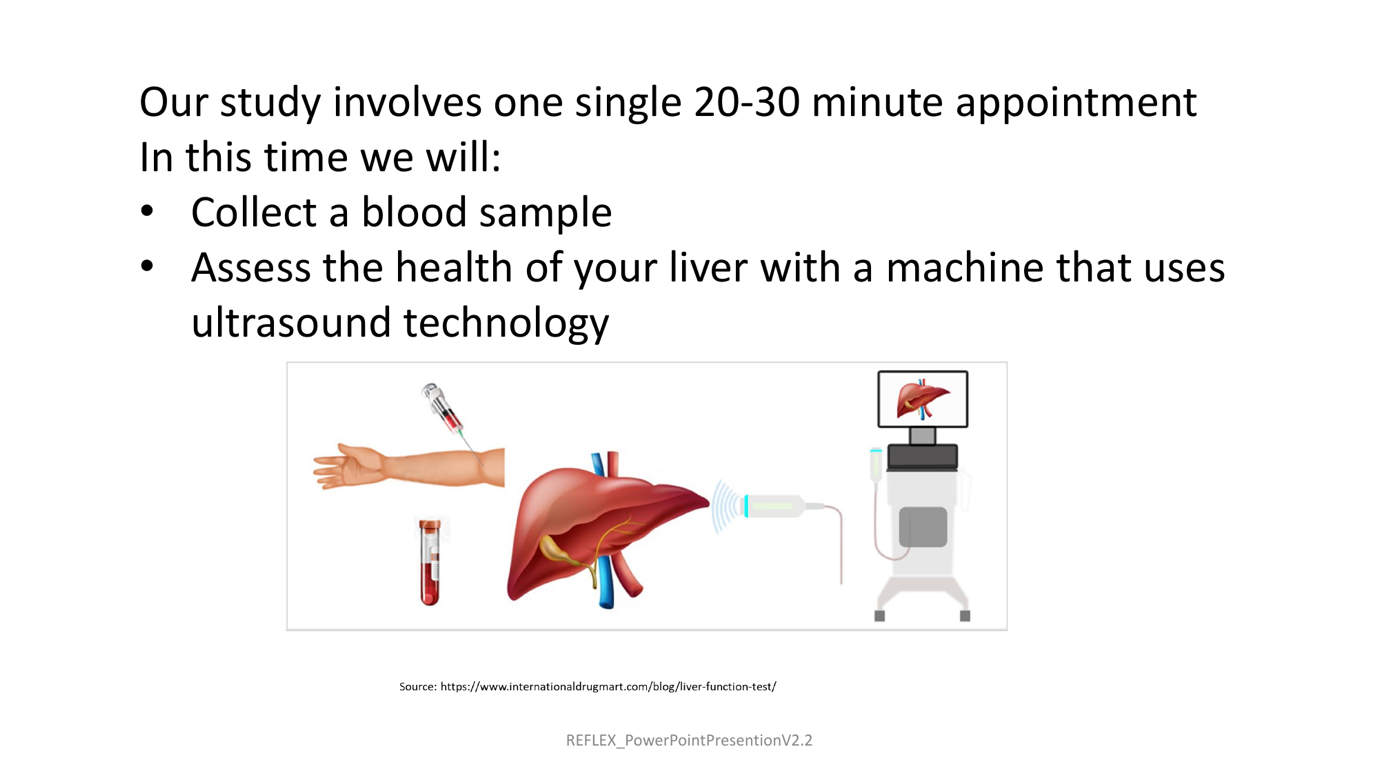

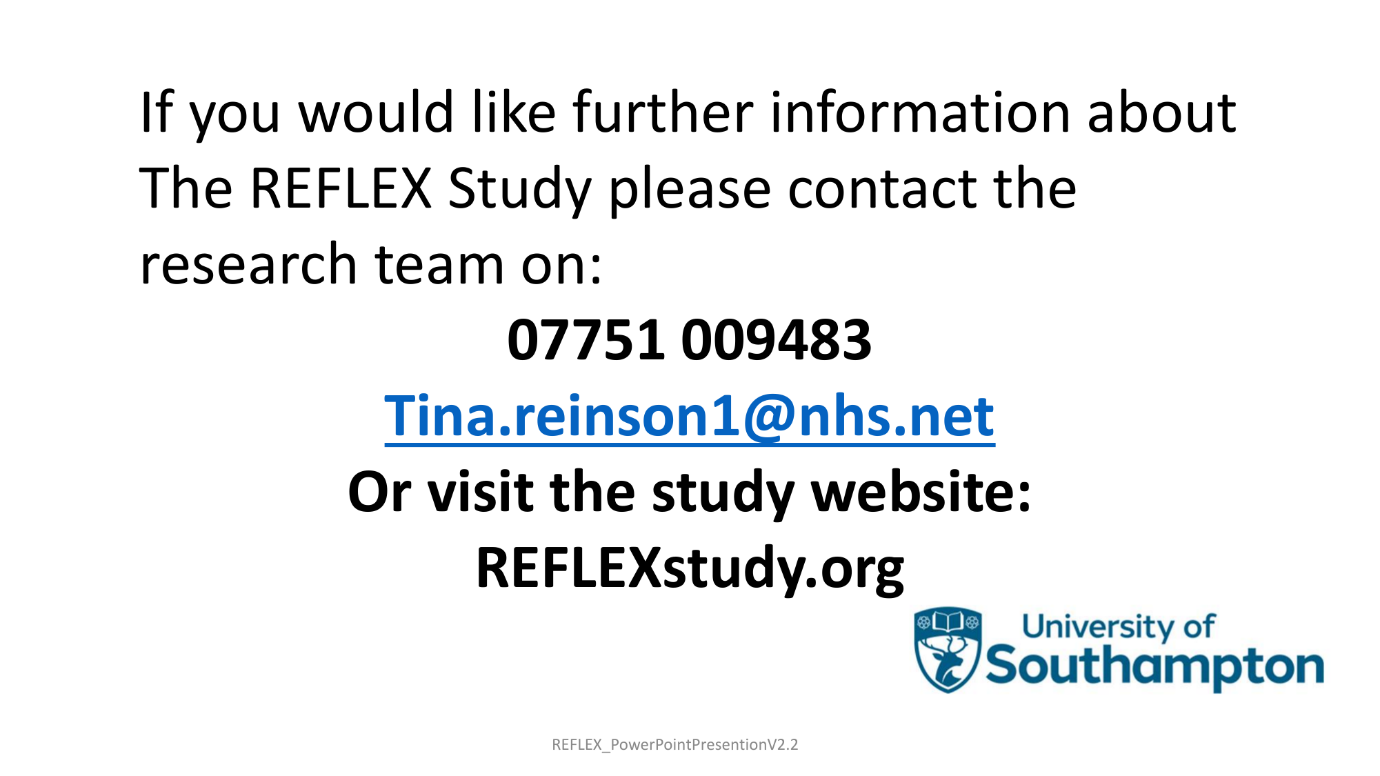


## Supplementary 11 – GP Website

<https://www.reflexstudy.org/wp-content/uploads/2023/08/gp_website.pdf>


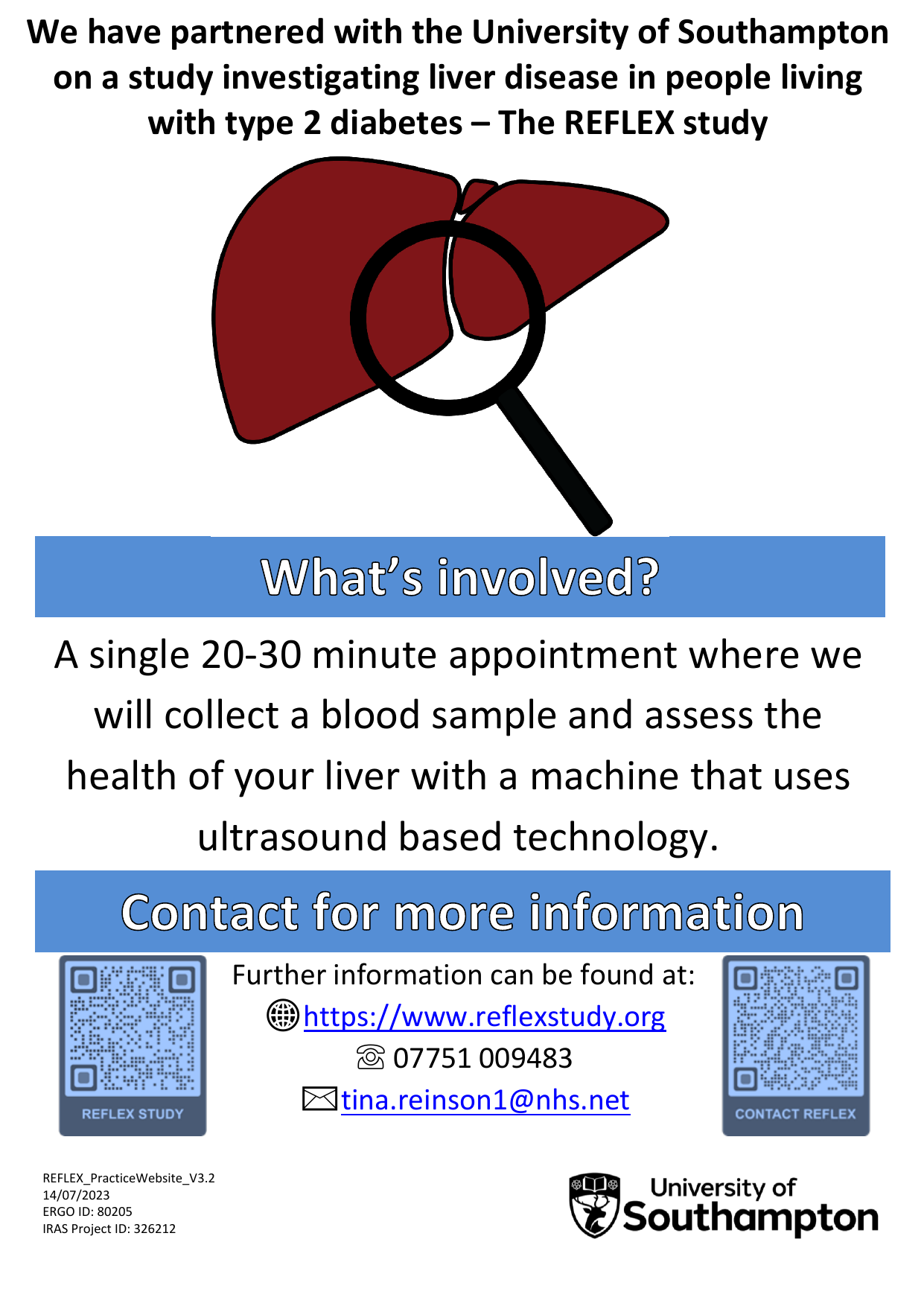


## Supplementary 12 – Flyer


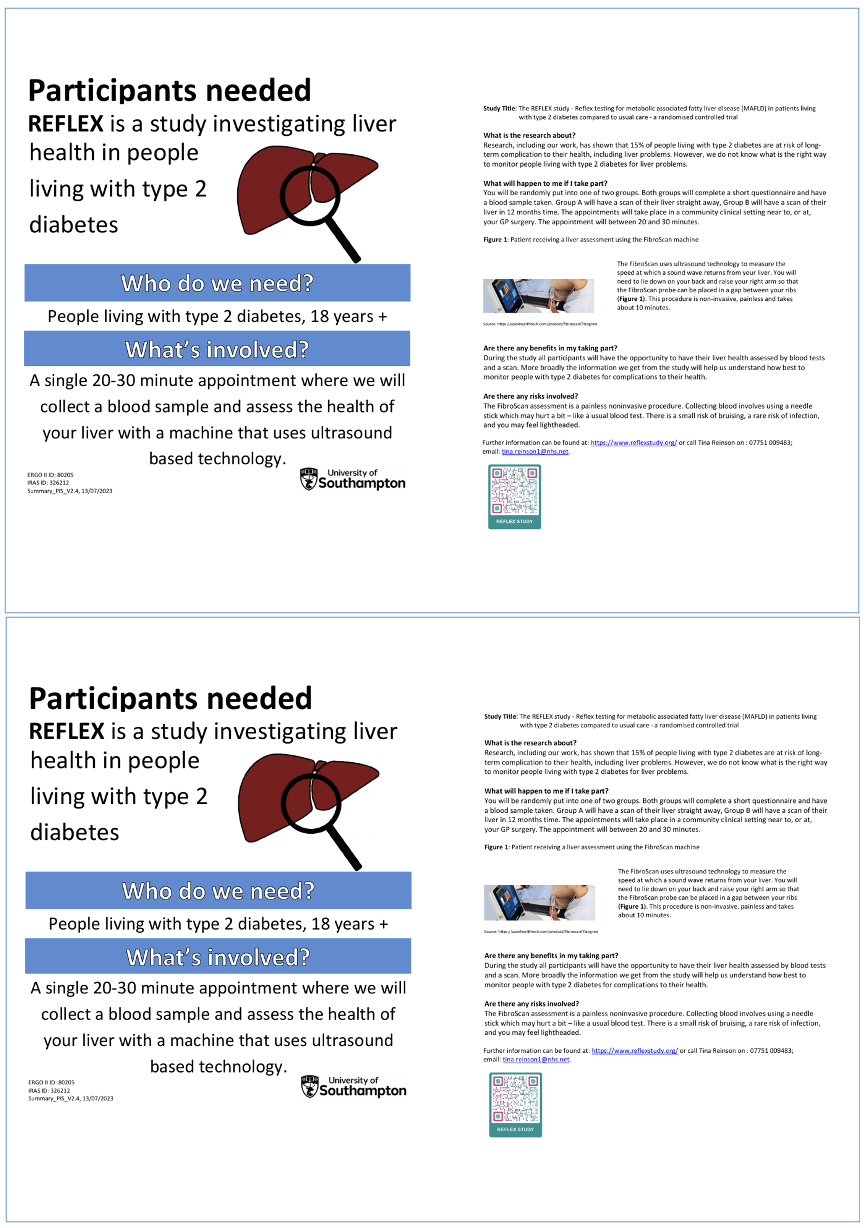
<https://www.reflexstudy.org/wp-content/uploads/2023/08/Appendix_3_SummaryPIS_V2.4_double_page.pdf>

## Supplementary 13 – Patient Letter

<https://www.reflexstudy.org/wp-content/uploads/2023/08/patient_letter.pdf>


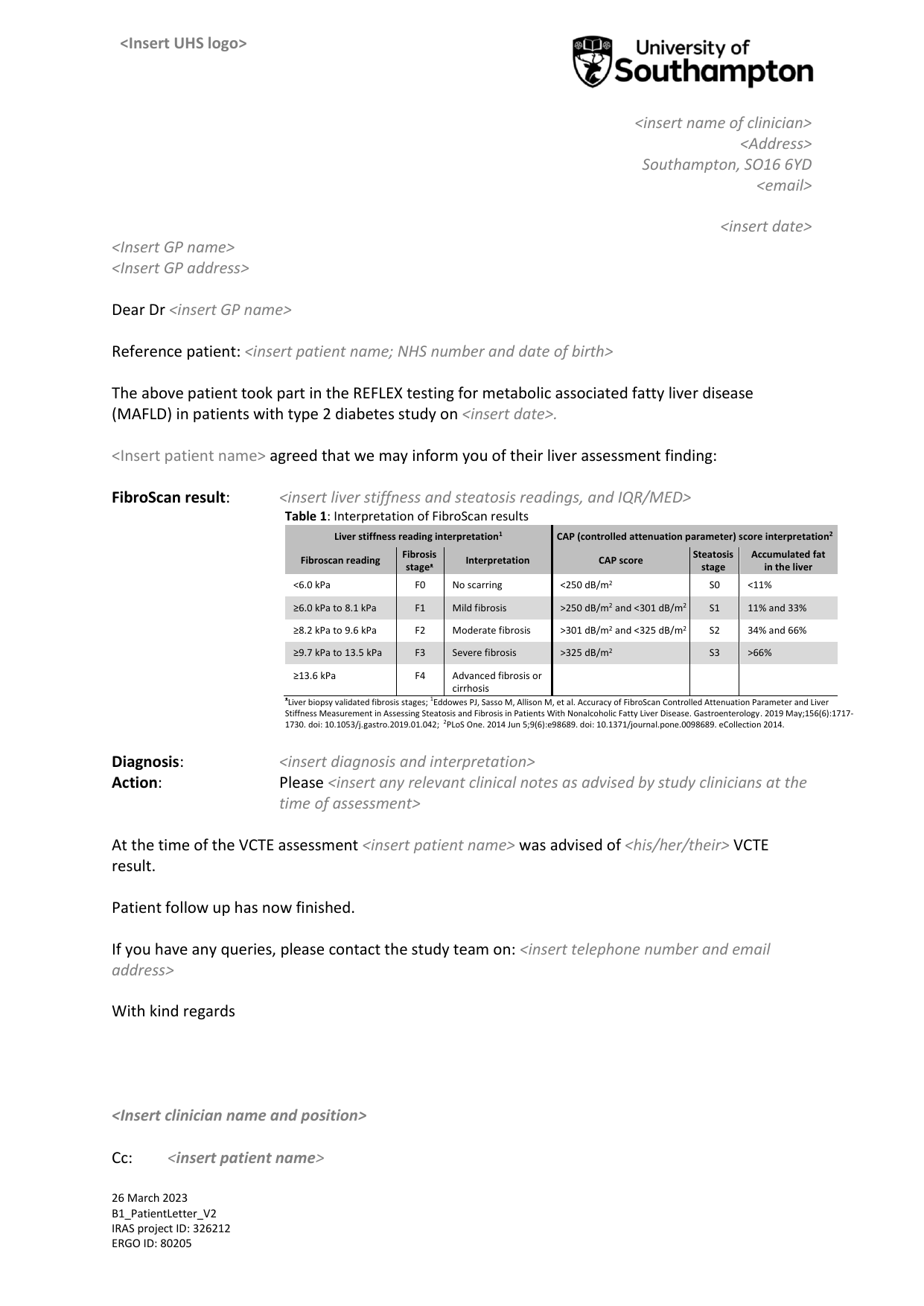


## Supplementary 14 – Missing data plan

**Sensitivity Analysis Plan to Manage Loss to Follow-Up (LTFU) in REFLEX**

**Objective:**
The purpose of this sensitivity analysis is to assess the robustness of the primary trial results to different assumptions about missing data caused by loss to follow-up (LTFU). The analysis will help determine how the outcomes would change under various scenarios related to the handling of missing data.

1. **Overview of Loss to Follow-Up and missing data**

Loss to follow-up (LTFU) can introduce bias if the participants lost to follow-up differ systematically from those who remain in the study. Sensitivity analysis will help address potential biases and provide a range of plausible outcomes based on different assumptions about missing data.

**Potential scenarios where missing data may affect our study**

1. A participant randomised to the intervention arm does not attend for liver assessment
2. A participant found to have a high liver stiffness does not attend for further clinical assessment and therefore the primary outcome (referral to HCC surveillance) is not assessed

**2. Primary Analysis Approach**

The primary analysis will use an **Intent-to-diagnose (ITD)** approach, including all randomised participants, regardless of whether they completed the study. For participants with missing outcome data due to LTFU, we will apply multiple imputation methods in the primary analysis to account for the uncertainty of missing data.

Alongside this we will present available data describing LTFU participants and compare them with participants who completed the study. This will be available as a supplementary table. The comparison will allow us to consider if LTFU was ‘non-random’ and how it may have influenced our conclusions.

**Multiple Imputation (MI)**

- **Description:** Multiple imputation will be used to impute missing values based on observed data, assuming that data are Missing at Random (MAR). Imputed datasets will be created using covariates that predict both missingness and the outcome.
- **Rationale:** MI allows us to handle uncertainty in the missing data and provides a range of plausible values, assuming the MAR assumption holds.
- **Interpretation:** Compare the results from MI with the complete case analysis. Large deviations would suggest sensitivity of the results to the MAR assumption.

**3. Sensitivity Analysis Approaches**

Subsequently several sensitivity analyses will be conducted to explore the impact of LTFU on the trial’s results. These will include:

**a. Complete Case Analysis**

- **Description:** Analyse only participants who complete the trial and for whom outcome data are available.
- **Rationale:** This represents a "best-case" scenario where LTFU is assumed to be random and does not introduce bias. However, if LTFU is not random, this could lead to biased results.
- **Interpretation:** The results from this analysis will be compared with the primary analysis (including MI for LTFU) to identify any major differences caused by the exclusion of participants lost to follow-up.

**b. Worst-Case/Best-Case Imputation**

- **‘Worst-Case’ Scenario:**
  - Assume that all participants lost to follow-up in the REFLEX group did not have the primary outcome (entry into HCC surveillance), while those in usual care did.
- **Best-Case Scenario:**
  - Assume the opposite: participants lost to follow-up in the REFLEX group were entered into HCC surveillance, while those in usual care were not.
- **Rationale:** These extreme-case analyses provide boundaries for the possible impact of missing data. If the conclusions remain similar to the primary analysis, the results are considered robust to LTFU.
- **Interpretation:** Significant changes between the worst-case/best-case scenario and the primary results would indicate that LTFU might have substantially influenced the trial’s findings.

**4. Assumptions and Limitations**

- **Missing at Random (MAR) vs. Missing Not at Random (MNAR):** The primary analysis assumes MAR, which means that the probability of being lost to follow-up depends only on observed characteristics. The sensitivity analyses (e.g., worst-case/best-case imputation) will allow us to assess how results change if data are MNAR.
- **Limitations:** Each method has its own limitations. Complete case analysis may introduce bias if LTFU is not random, and extreme-case scenarios may not reflect realistic assumptions. However, taken together, the sensitivity analyses will provide a range of outcomes under different assumptions.

**5. Reporting**

Results from the sensitivity analyses will be reported alongside the primary analysis. We will summarise:

- How each analysis affects the estimated treatment effect.
- Whether the conclusions of the trial (e.g., statistical significance, effect size) change under different assumptions about LTFU.
- Any substantial differences between the sensitivity analyses and the primary analysis, highlighting potential areas of concern regarding missing data.

**7. Managing missing data in the cost-effectiveness evaluation**

In our cost-effectiveness model the characteristics of the cohorts entering the model at time 0 will be based on ITD with MI for missing values. However, whether a patient in the model is engaged with HCC surveillance or other treatments will be determined by whether they engaged with liver assessment as part of the trial and usual care (if referred to hepatology services after assessment).

For example, if a participant is randomised but does not attend for liver assessment the stage of that participants liver disease will be determined by MI. But in the model (if via MI their liver stiffness is high) they will be assumed to have engaged with liver services so will not enter HCC surveillance or experience other benefits of engagement with care. Similarly, if a participant attends for liver assessment as part of the trial and has a high liver stiffness but does not engage with liver services they will not enter HCC surveillance or experience other benefits of engagement with care.

**8. Conclusion**

The sensitivity analysis will ensure that the trial's conclusions are robust to assumptions about missing data and LTFU. By considering multiple scenarios, the analysis will provide confidence in the validity of the results, or indicate areas where LTFU may have introduced bias. By taking these approaches we will ensure our cost-effectiveness results are cognisant with real-world levels of engagement with the liver diagnostic care cascade and doesn’t make the mistake of assuming 100% engagement.
