# Supplementary material for: Screening to identify people with type 2 diabetes at risk of liver cancer in primary care: a randomised controlled trial protocol
Source: BMJ Open. 2025 Mar 6;15(3):e088043. doi: 10.1136/bmjopen-2024-088043 (PMC11887308; doi:10.1136/bmjopen-2024-088043)
Supplement: Supplementary file 2 [file bmjopen-2024-088043.reviewer_comments.pdf]

PEER REVIEW HISTORY

BMJ Open publishes all reviews undertaken for accepted manuscripts. Reviewers are asked to complete a checklist review form and are provided with free text boxes to elaborate on their assessment. These free text comments are reproduced below.

ARTICLE DETAILS

Title (Provisional)

Screening to identify people with type 2 diabetes at risk of liver cancer in primary care - a randomised controlled trial PROTOCOL

Authors

Buchanan, Ryan; Reinson, Tina; Bilson, Josh; Woodland, Hazel; Nwoguh, Chinonso; Cooper, Keith; Harris, Scott; Malone, Karen; Byrne, C. D.

VERSION 1 - REVIEW

**Reviewer**	**1**
**Name**	**Ching, Siew Mooi**
**Affiliation**	**Universiti Putra Malaysia, Family Medicine**
**Date**	**05-Aug-2024**
**COI**	**nil**

Introduction:

1. Can you provide more context on the current screening practices for liver disease in people with T2DM in the UK?

2. How does this study build upon or differ from previous research on liver disease screening in T2DM populations?

3. What are the potential implications of this study for clinical practice and health policy?

5. Can you elaborate on the challenges of implementing widespread screening for liver disease in T2DM patients?

* The statement In the introduction section line25-32 on “Most patients with T2DM do not get tested for liver disease, despite their heightened risk because they are not assessed for the additional risk factors that are needed to qualify for testing. For example, LFTs are not part of an annual diabetes check up in the UK and may or may not be measured when patients are considered for statin treatment; liver ultrasound is not a routine test, and alcohol consumption is not accurately or consistently assessed in primary care.

" is contradicted with the statement stated in the method section line 12-1ine 18on "We will proceed

straight to an effectiveness evaluation rather than conducting a formal feasibility/pilot study because the components of the intervention (i.e. testing for liver disease in a primary care setting) are widely implemented and data such as the attrition rate from the diagnostic pathway is already known (see sample size section)"

Method:

1. How will you ensure standardization of VCTE measurements across different study sites?

2. Why* the HCC surveillance only take one year whereby the patient with NAFLD will take years to turn into HCC

2. What measures will be taken to blind outcome assessors to participants' group allocation?

3. How will you handle missing data in your analysis?

**Reviewer**	**2**
**Name**	**Saisho, Yoshifumi**
**Affiliation**	**Keio University School of Medicine Graduate School of Medicine Division of Rheumatology Department of Internal Medicine**
**Date**	**06-Aug-2024**
**COI**	**None declared.**

This is a protocol paper regarding screening to identify people with T2D at risk of liver cancer in primary care. This is a RCT to determine the effectiveness and cost-effectiveness of screening all adults with T2D to identify those at high risk of HCC. 320 participants will be randomized to screening. Screening includes measurement of bio-markers (ELF and Fib-4) and vibration controlled transient elastography. Another 320 participants will be randomized to usual care. Primary outcome is the proportion of participants in each arm who are referred into HCC surveillance over 12 months by specialist services.

The study is clinically important. The protocol is well designed and clearly described. There is no major issue in this protocol.

**Reviewer**	**3**
**Name**	**Reeves, Helen**
**Affiliation**	**Newcastle University Centre for Cancer**
**Date**	**29-Oct-2024**

COI none

I think this is an important study which will generate important data to inform early detection strategies for advanced liver disease in patients with diabetes, identifying those who may benefit from HCC surveillance.

However, I do have one major concern over the study design. 640 patients are to be recruited, with 320 in each group, with outcomes to compare between standard care and the study group, being detection of advanced liver disease and entry into a surveillance programme. I am unsure of the basis for the power calculations and the prediction of liver disease in the defined groups. The problem is the age entry criteria. This is standard at >18 years of age. But the average age of a person with fatty liver disease getting advanced fibrosis/cirrhosis, is between 50-60 years. The median age of a person with fatty liver disease cirrhosis getting an HCC is ~70 years. I would advise the authors consider this. If it is young keen people interested in research and highly motivated who take up the offer of this study, its unlikely the authors will find patients with chronic liver disease. I do appreciate that the authors are try not to 'only screen those with additional factors', but age is seriously important to consider here. I would suggest raising the age inclusion. I'm not a biostatistician, but to over 50 or even 60 years would seem sensible.

Reviewer 4

Name Dack, Kyle

Affiliation University of Bristol, MRC Integrative Epidemiology Unit

Date 28-Nov-2024

COI None

The protocol has a strong introduction which clearly demonstrates the clinical value and novelty of the study. The methods are thoroughly described and the statistical approach should achieve the study aims. I suggest a few small improvements.

Abstract

1. I suggest making the abstract more structured by adding subheadings – for example, some of those in the CONSORT for abstracts checklist (https://pmc.ncbi.nlm.nih.gov/articles/PMC2211558/). In particular, subheadings for participants, outcome, and intervention would be helpful for readers.

2. It is a little hard to understand just from the abstract what the comparison is: cirrhosis screening for patients with T2DM, vs screening only those with additional risk factors? A small explanation of "usual care" would remedy this.

Introduction

3. Formatting note: there is inconsistency with the referencing – sometimes a fullstop before a reference, sometimes a whitespace, and sometimes neither.

4. The study aim should include the outcome. The aim is currently stated as to record the performance of standard care vs a real-world pathway, but does not include what the performance metric is.

Methods

5. Arm 2 – standard care. It is not fully clear to me whether participants in this arm will have any further contact with the study after consenting to participate. Explicitly stating if they will/wont would clarify this.

6. The participant criteria excludes those who were evaluated for liver disease in the past 2 years, which is reasonable. However, in Arm 2, it is stated that as a part of standard care, individuals with "risk factors" may enter care pathways involving VCTE or biomarkers for fibrosis. Does this mean that the study will include very few participants with these risk factors? Because if I understand correctly, the pre-study standard care may have already led to these individuals being put on a screening pathway and therefore not meeting the inclusion criteria.

If so, this may be worth discussing in the main paper.

7. "We are concerned that the conduct of our study may increase liver disease diagnosed via usual care due to Hawthorne effect on participants randomised to usual care or on primary care physicians who are more likely to request testing because they are, as a consequence of participation, more aware of liver disease" Is it possible to access historical observational data on the rates of referral in the same study area, to evaluate whether there is a difference due to awareness?

VERSION 1 - AUTHOR RESPONSE

BMJ open response to comments:

Reviewer: 1
Dr. Siew Mooi Ching, Universiti Putra Malaysia, Universiti Putra malaysia
Comments to the Author:
Introduction:

1. Can you provide more context on the current screening practices for liver disease in people with T2DM in the UK?

Thank you for this important comment. See text in background see:
In addition to being recommended in the USA(25,26), screening for liver disease in patients with T2DM and obesity has recently been adopted as a national pilot in England that has been funded by the National Health Service England (NHSE) cancer service.(27) The national pilot uses a primary care

based search algorithm for T2DM as well as other risk factors for liver disease (such as hazardous alcohol consumption) and then invites patients into a cascade of non-invasive tests for fibrosis.

Whilst patients with T2DM are known to have an increased risk of fibrosis and cirrhosis(28) there is a lack of empirical evidence supporting implementation of this NHSE programme. Just three studies have tested a diagnostic pathway for liver disease against a contemporaneous control(29–31) and just one specifically focussed on liver disease in patients with T2DM(30).

The NHSE pilot is different from the current national (NICE) guidelines in the UK which recommends testing for liver disease is restricted to patients with risk factors for liver cirrhosis including a fatty liver on ultrasound imaging, abnormal liver enzyme levels and potentially harmful levels of alcohol consumption.(32)

2. How does this study build upon or differ from previous research on liver disease screening in T2DM populations?

Thank you – please see text in background:
Just three studies have tested a diagnostic pathway for liver disease against a contemporaneous control(29–31) and just one specifically focussed on liver disease in patients with T2DM(30).

3. What are the potential implications of this study for clinical practice and health policy?

Thank you please see following text in discussion:
If effective the trial would provide evidence toward justifying widespread screening in an enormous, and growing proportion of the global population with a knock-reduction in liver death. If not effective, it could prevent further roll out of a massive, costly programme of work that will have significant resource implications for health service systems. Looking forward the trial will also quantify the effect size required and suitable pricing for novel anti-fibrotic therapies to meet cost-effectiveness thresholds.

5. Can you elaborate on the challenges of implementing widespread screening for liver disease in T2DM patients?
* The statement In the introduction section line25-32 on "Most patients with T2DM do not get tested for liver disease, despite their heightened risk because they are not assessed for the additional risk factors that are needed to qualify for testing. For example, LFTs are not part of an annual diabetes check up in the UK and may or may not be measured when patients are considered for statin treatment; liver ultrasound is not a routine test, and alcohol consumption is not accurately or consistently assessed in primary care.
" is contradicted with the statement stated in the method section line 12-1ine 18on "We will proceed
straight to an effectiveness evaluation rather than conducting a formal feasibility/pilot study because the components of the intervention (i.e. testing for liver disease in a primary care setting) are widely implemented and data such as the attrition rate from the diagnostic pathway is already known (see sample size section)"

Thank you for identifying this contradiction the text has been amended as follows to give clarity:
"We will proceed straight to an effectiveness evaluation rather than conducting a formal feasibility/pilot study because the components of the intervention (used in testing for liver disease in

patients with standard risk factors (i.e. abnormal blood results or harmful alcohol consumption) are widely implemented and data such as the attrition rate from the diagnostic pathway is already known"

1. How will you ensure standardization of VCTE measurements across different study sites?

See text in methods:
"VCTE will be performed by an experienced single operator after a minimum of a 3 hour fast and previously published criteria for a valid reading will be applied to each participant.(40)"

2. Why* the HCC surveillance only take one year whereby the patient with NAFLD will take years to turn into HCC

The study follow-up period is one year because the primary outcome is entry into HCC surveillance. Incident HCC in our cohort will then be subject to Markov modelling we a life-time time horizon. This will allow our short (and feasible) study to make conclusions based on cancer outcomes.

2. What measures will be taken to blind outcome assessors to participants' group allocation?

The clinicians who determine the primary outcome (entry into HCC surveillance or not) are independent health-care practitioners (e.g. hepatology nurse specialist) who are not involved in the study. By the nature of the intervention participants will not be blinded. However, since the outcome is objective and determined by independent clinicians we anticipate the risk that this introduces bias to be minimal. See text:

"As an unblinded trial it is important our primary outcome is as objective as possible and independent of the research team. In both study arms patients with high liver stiffness measurements will be referred to nearby hepatology services (with thresholds defined by local practice). Via usual care an independent local clinician will then assess the severity of liver disease. In real-world practice this may include history, examination, no further tests or repeat VCTE, additional tests for fibrosis and in some case liver biopsy."

3. How will you handle missing data in your analysis?

Thank you for this important point. We have a missing data analysis plan (see supplementary material page 22) and amended text in main manuscript. We hope this is acceptable.

"Loss to follow up (LTFU) and missing data will be managed in accordance for our missing data management plan (see supplementary material)."

Reviewer: 2

Comments to the Author:
This is a protocol paper regarding screening to identify people with T2D at risk of liver cancer in primary care. This is a RCT to determine the effectiveness and cost-effectiveness of

screening all adults with T2D to identify those at high risk of HCC. 320 participants will be randomized to screening. Screening includes measurement of bio-markers (ELF and Fib-4) and vibration controlled transient elastography. Another 320 participants will be randomized to usual care. Primary outcome is the proportion of participants in each arm who are referred into HCC surveillance over 12 months by specialist services.
The study is clinically important. The protocol is well designed and clearly described. There is no major issue in this protocol.

Thank you for your positive comments and the time you have taken to review our study protocol.

Reviewer: 3
Helen Reeves, Newcastle University Centre for Cancer
Comments to the Author:
I think this is an important study which will generate important data to inform early detection strategies for advanced liver disease in patients with diabetes, identifying those who may benefit from HCC surveillance.

However, I do have one major concern over the study design. 640 patients are to be recruited, with 320 in each group, with outcomes to compare between standard care and the study group, being detection of advanced liver disease and entry into a surveillance programme. I am unsure of the basis for the power calculations and the prediction of liver disease in the defined groups. The problem is the age entry criteria. This is standard at >18 years of age. But the average age of a person with fatty liver disease getting advanced fibrosis/cirrhosis, is between 50-60 years. The median age of a person with fatty liver disease cirrhosis getting an HCC is ~70 years. I would advise the authors consider this. If it is young keen people interested in research and highly motivated who take up the offer of this study, its unlikely the authors will find patients with chronic liver disease. I do appreciate that the authors are try not to 'only screen those with additional factors', but age is seriously important to consider here. I would suggest raising the age inclusion. I'm not a biostatistician, but to over 50 or even 60 years would seem sensible.

Thank you for this important point. As you correctly point out liver disease to associated with increasing age in patients with T2DM. In this study we wanted to determine the effectiveness and cost-effectiveness of a blanket offer of testing for liver disease to adults with T2DM (who will mostly be older(>50% aged over 65 years in UK[1])) in part because this (without a lower limit age restriction) is what is being considered/piloted nationally by the NHS in England. If, in our study a relatively young cohort comes forward then that is (in our view) a reasonable representation of what would happen in real-world practice – and therefore a strength. Additionally, having a younger cohort isn't implicitly a bad thing from a cost-effectiveness perspective. For those (relatively few) young people with liver disease there will be more time to 'gain' quality of life in our cost-effectiveness modelling. In effect a younger cohort with less liver disease could be a more cost-effective cohort to work with.

*Please also note – in the time since we submitted this protocol we have begun recruitment. Initial participants have a median age of 69 years.

1. https://www.statista.com/statistics/387343/individuals-with-type-2-diabetes-by-age-in-england/

Reviewer: 4
Dr. Kyle Dack, University of Bristol

Comments to the Author:
The protocol has a strong introduction which clearly demonstrates the clinical value and novelty of the study. The methods are thoroughly described and the statistical approach should achieve the study aims. I suggest a few small improvements.

Abstract
1. I suggest making the abstract more structured by adding subheadings – for example, some of those in the CONSORT for abstracts checklist (https://pmc.ncbi.nlm.nih.gov/articles/PMC2211558/). In particular, subheadings for participants, outcome, and intervention would be helpful for readers.

Thank you for this important point. Unfortunately, we are constrained by the formatting guidelines from the journal.

2. It is a little hard to understand just from the abstract what the comparison is: cirrhosis screening for patients with T2DM, vs screening only those with additional risk factors? A small explanation of "usual care" would remedy this.

Thank you see amended text in abstract.
"A multi-site, unblinded individual randomised controlled trial comparing screening for liver cirrhosis in people with T2DM against usual care - where additional risk factors are needed to qualify for screening."

Introduction
3. Formatting note: there is inconsistency with the referencing – sometimes a fullstop before a reference, sometimes a whitespace, and sometimes neither.

Thank you – now consistent throughout

4. The study aim should include the outcome. The aim is currently stated as to record the performance of standard care vs a real-world pathway, but does not include what the performance metric is.

Point well made – thank you – see amendment, we hope this is acceptable.
"This study protocol describes a randomised controlled trial with a nested cost-effectiveness evaluation. The study aims to compare the number of participants referred for HCC surveillance between an intervention where patients with T2DM are universally offered screening for liver disease against usual care."

Methods
5. Arm 2 – standard care. It is not fully clear to me whether participants in this arm will have any further contact with the study after consenting to participate. Explicitly stating if they will/wont would clarify this.

Thank you see amended text.

6. The participant criteria excludes those who were evaluated for liver disease in the past 2 years, which is reasonable. However, in Arm 2, it is stated that as a part of standard care, individuals with “risk factors” may enter care pathways involving VCTE or biomarkers for fibrosis. Does this mean that the study will include very few participants with these risk factors? Because if I understand correctly, the pre-study standard care may have already led to these individuals being put on a screening pathway and therefore not meeting the inclusion criteria.
If so, this may be worth discussing in the main paper.

Thank you for raising this. To clarify – those participants in the usual care arm may be assessed for liver disease during the remote up interval (12 months) but existing guidance means that it is only likely to be those with a risk factor for liver disease over and above simply having T2DM e.g. an abnormal ALT.

The exclusion criteria you refer to (excluding those who have been screened in the past 2 years) applies to both arms. This is to represent what the testing intervention would look like in practice i.e. patients with T2DM who have been recently assessed for liver disease won’t be eligible for reassessment during a pre-specified interval. Currently 2 years is used in most services for populations who are currently eligible for screening - although the evidence base underlying this duration isn’t robust.

7. “We are concerned that the conduct of our study may increase liver disease diagnosed via usual care due to Hawthorne effect on participants randomised to usual care or on primary care physicians who are more likely to request testing because they are, as a consequence of participation, more aware of liver disease” Is it possible to access historical observational data on the rates of referral in the same study area, to evaluate whether there is a difference due to awareness?

Good point. Yes, we have an idea about the background testing rate in patients with T2DM in our region. We have used it to support our sample size calculation by giving us an idea about what is likely to happen in our usual care arm (see below). As you suggest we can use that rate to look for evidence of behaviour change due to the Hawthorne effect or other phenomena in our study. However, as this isn’t contemporaneous and is observational data it will be difficult to draw causal inferences.

Below is the data we used to calculate what is likely to happen in our control arm. We knew that roughly 4% of patients with T2DM get assessed for liver disease in part of our region. We doubled this rate to cover us for any increased in background testing during the study.

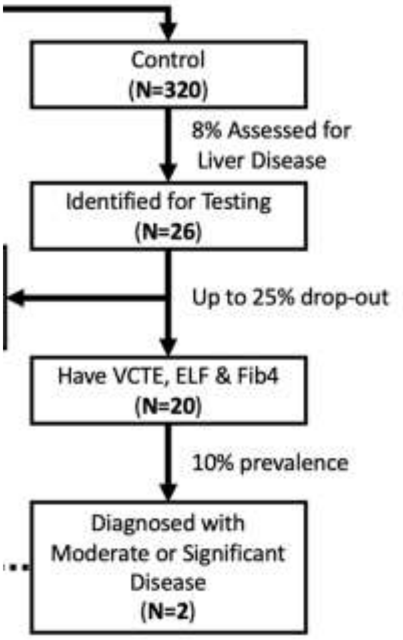

Background rate of testing ~4% - we doubled this to ensure we have enough power
